# Attribution of Ghrelin to Cancer; Attempts to Unravel an Apparent Controversy

**DOI:** 10.3389/fonc.2019.01014

**Published:** 2019-10-16

**Authors:** Saeed Soleyman-Jahi, Fatemeh Sadeghi, Amin Pastaki Khoshbin, Leila Khani, Venus Roosta, Kazem Zendehdel

**Affiliations:** ^1^Division of Gastroenterology, School of Medicine, Washington University in St. Louis, St. Louis, MO, United States; ^2^Cancer Immunology Project, Universal Scientific Education and Research Network, St. Louis, MO, United States; ^3^Cancer Research Center, Cancer Institute of Iran, Tehran University of Medical Sciences, Tehran, Iran; ^4^Cancer Immunology Project, Universal Scientific Education and Research Network, Tehran, Iran; ^5^Department of Immunology, School of Medicine, Iran University of Medical Sciences, Tehran, Iran; ^6^School of Medicine, Tehran University of Medical Sciences, Tehran, Iran

**Keywords:** ghrelin, cancer, carcinogenesis, prognosis, orexigenic

## Abstract

Ghrelin is an endogenous peptide hormone mainly produced in the stomach. It has been known to regulate energy homeostasis, stimulate secretion of growth hormone, and mediate many other physiologic effects. Various effects attributed to ghrelin contribute to many aspects of cancer development and progression. Accordingly, a large body of evidence has emerged about the association of ghrelin with several types of cancer in scales of cell-line, animal, and human studies. However, existing data are controversial. This controversy occurs in two main domains: one is the controversial results in local effects of ghrelin on different types of human cancer cell-lines; the second is the apparent disagreement in the results of *in-vitro* and clinical studies that investigated ghrelin association to one type of cancer. These inconsistencies have hampered the indications to consider ghrelin as a potential tumor biomarker or therapeutic agent in cancer patients. Previous studies have reviewed different parts of current literature about the ghrelin-cancer relationship. Although they have highlighted these controversial results in various ways, no specific recommendations have been given to address it. In this study, we comprehensively reviewed *in-vitro, in-vivo*, and clinical studies and attempted to use the following approaches to unravel the inconsistencies detected: (a) to distinguish local and systemic effects of ghrelin in interpreting its summary clinical role in each cancer; (b) scrutinizing factors that regulate local effects of ghrelin and could justify different effects of ghrelin on different cancer cell-lines. These approaches could have notable implications for future *in-vitro* and clinical studies.

## Introduction

**Figure 1:**
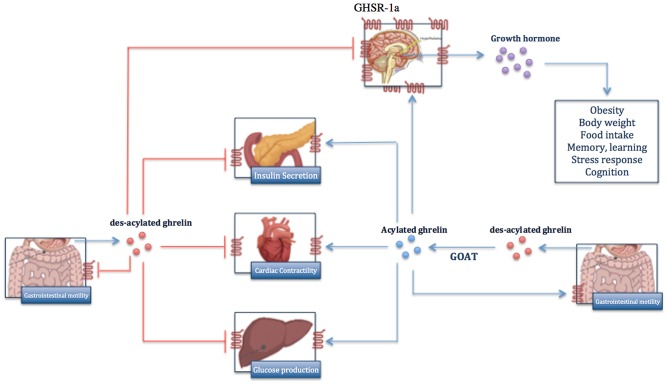
Overview of biological functions of ghrelin in body. Ghrelin is mainly produced in des-acylated form by gastric oxyntic gland and is acylated to active ghrelin by GOAT enzyme. Ghrelin exerts its biological effects through binding to GHR-1, which is expressed predominantly on hypothalamus and less on other organs. In contrast, des-acylated ghrelin functions as ghrelin antagonist and inhibits the function of ghrelin.

**Figure 2:**
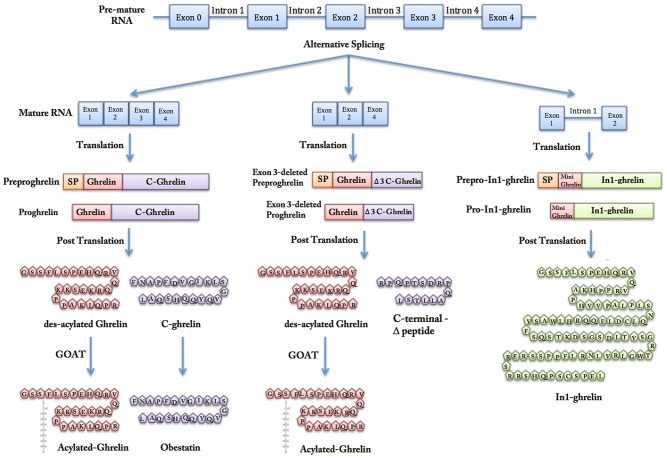
Gene transcription, alternative splicing, and post-translational modifications of ghrelin. Ghrelin is encoded by GHRL gene, which also can produce peptides other than native ghrelin through alternative splicing. Exon 3-deleted peptide lacks the exon number 3 and In1-ghrelin is product of a messenger RNA, which retains the intron-1 transcript of GHRL gene. The initial form of each peptide contains a N-terminal signal peptide that is cleaved by signal peptidase in the endoplasmic reticulum and gives rise to proghrelin, exon 3-deleted proghrelin, and pro-In1-ghrelin. By further post-translational processing, proghrelin is cleaved to produce different peptides.

Ghrelin is a peptide hormone that was discovered as an intrinsic ligand for growth hormone secretagogue receptor (GHSR) (1). Ghrelin is mainly produced in des-acylated form by the gastric oxyntic gland (Figure 1) (2–5). Other tissues including the lungs, kidneys, intestines, pancreas, gonads, pituitary, and hypothalamus also produce lower levels of ghrelin (4–8). Ghrelin is encoded by the *GHRL* gene, which is located in the short arm of chromosome 3 (3p25-26) (Figure 2) (9). The initial *GHRL* gene product is a 117-amino acid pre-proprotein, called pre-proghrelin. Pre-proghrelin contains a 23-amino acid N-terminal signal peptide that is cleaved by signal peptidase in the endoplasmic reticulum (5, 9). The remaining proghrelin peptide is then split into 28-amino acid ghrelin and 66-amino acid C-ghrelin peptides, by a prohormone convertase. Obestatin is another regulatory hormone, which is generated by further processing of C-ghrelin (5, 10).

Alternative splicing of *GHRL* gene transcript leads to synthesis of peptides other than native ghrelin, C-ghrelin, and Obestatin (5, 11, 12). Exon 3-deleted pre-proghrelin and prepro-In1-ghrelin are two products of alternative splicing that will be discussed in more details later in this paper. Exon 3-deleted preproghrelin apparently undergoes a processing similar to that of preproghrelin, which gives rise to native ghrelin and a unique carboxy-terminal peptide different from C-ghrelin (11). Prepro-In1-ghrelin is the product of a messenger RNA, which retains the intron1 transcript of the *GHRL* gene. After removal of the signal peptide, the unique In1-ghrelin produced is larger than the native ghrelin (12).

Total native ghrelin consists of acylated and des-acylated ghrelins. Both forms are found in ghrelin-producing cells as well as in the circulation (13). Acylation is a distinct post-translational modification mediated by the enzyme Ghrelin-O-Acyltransferase (GOAT) (5). During acylation, a fatty acid chain, mostly octanoyl coenzyme A, is attached to the Serine 3 residue of proghrelin (5). GOAT is present in ghrelin-producing cells and is predominantly located in the endoplasmic reticulum membrane (14, 15). Acylation seems to be a key regulatory mechanism of ghrelin functions, since physiological concentrations of des-acylated ghrelin cannot directly activate the main known ghrelin receptor, GHS-R1a (16). Des-acylated from constitutes approximately 90% of total ghrelin in the circulation (17). Notably higher concentrations of des-acylated ghrelin in the circulation may be explained by: a larger proportion of ghrelin being secreted in des-acylated form, conversion of acylated ghrelin to des-acylated form in the circulation by serum esterase, and more stability of des-acylated ghrelin in the circulation (5, 18). The half-life of circulating des-acylated ghrelin is consistently reported to be higher than the acylated form (19–21). In one study, elimination half-lives of total and acylated ghrelin in human plasma were ~35 and 10 min, respectively (19).

The current body of evidence supports notable complexity in ghrelin axis, fed by both genetic and functional multiplicities (22). Ghrelin exerts its paracrine and endocrine functions through interactions with identified and unidentified receptors on target cells (5). Ghrelin receptors characterized so far are two splice variants of GHSR, type 1a (GHSR1a) and 1b (GHSR1b), which are G protein-coupled and widely expressed. GHS-R1a, which is considered the main functional receptor that mediates most of the physiologic effects, is a transmembrane G-protein coupled receptor (13). Acylated ghrelin is the only form of ghrelin that can activate GHS-R1a (23, 24). The acyl group is required for the conformational changes in ghrelin peptide, which leads to activation of GHS-R1a (23). Upon activation of GHS-R1a, a Gq protein-coupled phospholipase-3/inositol-3-phosphate signaling mediates calcium release from endoplasmic reticulum (9). GHSR1b is a truncated splice variant. Although the exact function of GHS-R1b is yet to be described, it may modulate GHS-R1a signaling by forming GHS-R1a/R1b heterodimers (25, 26). It is also believed to prevent GHSR1a transfer from endoplasmic reticulum and regulate its expression on the cell surface (25). Despite diverse physiologic and pathologic functions proposed so far for des-acylated ghrelin, its cognate receptor remains to be discovered (16, 27).

Regulation of fat metabolism, gut motility, secretion of gastric acid, and modulation of sleep are among paracrine biological effects (28–30). Modulation of appetite (30), anti-inflammatory effects (31), and regulating endocrine function constitute main systemic effects. Given these effects, ghrelin can potentially contribute to cancer development and behavior. Proliferative roles of ghrelin have been studied in hepatoma (32), leukemia (33), colorectal (34, 35), pancreas (4), prostate (36), and breast (11, 12) tumor cell lines, mainly through mechanisms other than ghrelin-growth hormone axis. Ghrelin also promoted migration and invasion in gastric (37) and pancreatic adenocarcinoma (38) via GHSR/NF-kB and GHSR/PI3K/Akt signaling pathways, respectively. Likewise, ghrelin inhibition significantly blocked the migration and invasion of human colon cancer cell lines (35). Furthermore, ghrelin could augment angiogenesis via ERK2 signaling pathway (39).

Chronic and local inflammation are linked to tumorigenesis and cancer progression (40, 41). Therefore, ghrelin can be linked to cancer through its anti-inflammatory effects. Ghrelin administration significantly retarded inflammation-associated colon carcinogenesis (42). Furthermore, exogenous ghrelin hampered systemic inflammatory response in esophageal cancer patients (43).

Other reports indicate the controversial attribution of both local and systemic effects of ghrelin to cancer biology. Exogenous ghrelin had neutral effects on proliferation and invasion of some cancer cell lines. Likewise, ghrelin was not associated with prognosis of lung cancer (44) or aggressiveness of esophageal cancer (45).

Beside controversies in ghrelin contribution to different cancers, inconsistencies exist between local *in-vitro* and summary clinical effects of ghrelin on the same type of cancer. Although higher blood ghrelin was associated with decreased incidence of colorectal cancer (46), higher local ghrelin expression in tumor tissue correlated with more aggressive tumors (35). Likewise, although *in-vitro* studies reported stimulatory effects of ghrelin on proliferation and invasion of gastric cancer cell lines (37, 47), the clinical studies linked ghrelin to a reduced incidence (48, 49), and better prognosis (50). Finally, inconsistencies even exist in the prognostic roles of serum ghrelin measured before or after gastrectomy (50).

Several authors have attempted to summarize ghrelin attributions to cancer (28, 51–57). A review focused mainly on local effects and expression of ghrelin in different types of cancers (52). The majority of studies showed the carcinogenic effects of ghrelin on cancer cell-lines. Clinical studies reporting prognostic/therapeutic roles of ghrelin were not included. Another review focused only on human and animal studies. *In-vitro* studies were excluded. These studies showed a null or inverse association between ghrelin and risk/progression of most cancers (53), highlighting controversies between their conclusion and previous reviews. A more recent review included data from *in-vitro* and clinical studies as well as The Cancer Genome Atlas (TCGA) to investigate the role of ghrelin and its receptor in cancer (22). The authors underscored differential expression of ghrelin receptors as a potential explanation for the controversial role of ghrelin in different cancers.

A comprehensive consideration of *in-vitro* and systemic effects of ghrelin in cancer is missing from previous reviews. These also did not provide explanations for apparent controversies between *in-vitro* studies on different cancers, as well as between *in-vitro* and clinical studies of the same type of cancer. Finally, they did not delineate the effects of ghrelin on carcinogenesis and prognosis. We considered specific approaches in this review to address these inconsistencies.

## Breast Cancer

### Attribution of Ghrelin Axis to Cancer Pathogenesis and Differentiation

#### Expression of Native Ghrelin Peptide

Among 144 patients with invasive breast cancer, 88.2% had ghrelin positive tumor cells (Table 1; Supplementary Table 1). Expression of ghrelin was linked to estrogen receptor (ER) status, but not to the status of progesterone receptor (PR) and HER2/neu receptors (96). Authors reported an inverse association of ghrelin expression with tumor size, tumor grade, and expression of the proliferation marker Ki-67. Similarly, 81.2% of 197 male patients with breast cancer had ghrelin positive tumor cells. The intensity of ghrelin staining was not uniform in different parts within a tumor, suggesting a shift from production of native ghrelin to other variants of ghrelin in malignant conditions (97). Non-malignant breast tissue expressed no ghrelin (58), or lower levels of ghrelin compared to malignant tissue (11). Quantification of ghrelin at transcript level did not show a significant difference between normal and malignant breast tissues (12).

**Table 1:** Summary of *in-vitro, in-vivo*, and clinical data concerning the expression of ghrelin-axis in different types of cancer.

	**Ghrelin**			**GHSR-1a**		**GHSR-1b**		**Other**	**References**
	**mRNA**	**Local protein level**	**Blood/cell culture media protein concentration**	**mRNA**	**Protein**	**mRNA**	**Protein**		
Breast cancer									
Cancer cell lines									
T47D	pG +, ex 3-del +			–, +		+			(11, 29)
MDA-MB231	pG +, ex 3-del +, native –, In-1 +			–, +, –		+, +		GOAT +	(11, 12, 29)
MCF7	pG +, ex 3-del +			–, +		+			(11, 29)
MB-435	pG +, ex 3-del +			–, +		+			(11)
Human samples									
Normal	Native +, In-1 +	Native –, aG +, ex 3-del +		+	++, +	–	–	GOAT +	(11, 12, 58)
Benign		Native –			+				(58)
Carcinoma	Native +, In-1 ++, In-1 +	Native +, aG ++, ex 3-del ++	Cachectic > non- cachectic (total); CADS > non-CADS (total)	+	+, +	+	+	GOAT ++	(11, 12, 58–61)
Colorectal cancer									
Human samples									
Normal		Native +	Carcinoma < normal (2), Carcinoma > normal, Cachectic > non- cachectic (total) (2)		++		+	*GHRLOS LncRNA++*	(35)
Carcinoma		Native ++			+		++	*GHRLOS LncRNA+*	(35, 61–64)
Gastric cancer									
Cancer cell-lines									
AGS	Native –			+, +^*^		+			(37, 47)
SGC7901				++^*^					(37)
Human samples									
Normal	Native ++	Native ++	Neuroendocrine = normal (total), Adenocarcinoma < normal (total) (3)	+^*^					(65, 66)
Adenocarcinoma	Native +	Native +		++^*^					(48, 49, 65–68)
Neuroendocrine									
Esophageal cancer									
Human samples									
Normal			Normal > adenocarcinoma (total) Normal > SCC (total) Normal > EGJA (total)	+					(69)
Barrett				++					(69)
Adenocarcinoma	Native –	Native –							(70, 71)
SCC									(72)
EGJA									(49)
Lung cancer									
Cancer cell lines									
CALU-1 (non-endocrine)				–					(73)
H1650 and HCC827/GR (drug-resistant NSCLC)				++^*^	++^*^				(74)
HCC827 (non-drug resistant NSCLC)				+^*^	+^*^				(74)
H345 (neuro-endocrine)	Native +	Native –		–					(75)
Human samples									
SCLC			SCLC = normal SCLC > normal (2) Cachectic > non-cachectic NSCLC = normal NSCLC > normal (2)						(44, 76)
NSCLC						+			(44, 76, 77)
Adenocarcinoma	Native +	Native –		–					(75)
SCC	Native +	Native –		+					(75)
Neuroendocrine	Native +	Native +		+					(75)
Prostate cancer									
Cancer cell-lines									
DU145	Native ++, native –, native +, In-1 +	Native –	Native –	+, +		+, +			(78–80)
LNCaP	Native +, native –, aG +, ex 3-del +, native ++, In−1 +	Native –	Native –	+, –		+, –			(36, 78–80)
PC3	Native + (3), aG ++, ex 3-del ++, In-1 ++		Native –	+, –		++, –			(36, 78–80)
ALVA41	Native +, In-1 +			++		+			(78, 79)
22Rv1	Native +, In-1 +			+		+			(78, 79)
VCaP	Native +, In-1 +								(79)
Normal cell line	Native –, native +, In-1+			+		–			(78, 79)
Normal	Native +, In-1 +		Carcinoma = benign (total) (2), Carcinoma > benign (aG), Carcinoma > normal (aG), Carcinoma = Normal (aG), Carcinoma > normal (In-1)	–		–			(78)
Benign	Native +	Native –, Native +, ex 3-del +				+			(36, 80)
Carcinoma	Native + (2), In-1 ++	native –, native ++, ex 3-del ++		– (2)		– (2)		Carcinoma > normal (GOAT)	(36, 78, 80–83)
Pancreatic cancer									
Cancer cell lines									
PANC1	Native +	Native –		+	+	+	+		(38)
MIAPaCa2	Native –	Native –		+	+	+	+		(38)
BxPC3	Native –	Native –		+	+	+	+		(38)
Capan2	Native –	Native –		+	+	+	+		(38)
Human samples									
Normal			Neuroendocrine = normal (total) (2)						
Neuroendocrine tumor	Native +	Native +		+^*^	+^*^				(68, 84)
Clear cell subtype of renal cell carcinoma (ccRCC)									
Cancer cell-lines									
786-0		Native ++			+				(85)
ACHN		Native +			+				(85)
A-498		Native +			+				(85)
769-P		Native +			++				(85)
A-704		Native +			+				(85)
Human samples									
Normal		Native +							(85)
Carcinoma		Native ++							(85)
Central nervous system cancer									
Cancer cell lines									
Astrocytoma (CCF-STTG1, U-87, U-118, SW1088)	Native +	Native +	Native+	+	+				(86)
Glioma: C6 (rat), U251(human)				+	+	+	+		(87)
Normal astrocytes	Native –/+	Native –/+	Native –/+	–/+	–/+				(86)
Human samples									
Glioblastom		Native (38/39)			+ (38/39)				(88)
Anaplastic astrocytoma		Native (12/13)			+ (5/13)				(88)
Diffuse astrocytoma		Native (2/11)			+ (1/11)				(88)
Neurofibromatosis					+^*^				(89)
Ovarian cancer									
Cancer cell lines									
HO-8910				+^*^					(90)
Human samples									
Benign		Native: benign > borderline > malignant							(91)
Borderline									
Malignant									
Endometrial cancer									
Human samples									
Normal		Native +			+				(92)
Cancer		Native + (2)			+				(92, 93)
Oral squamous cell carcinoma									
Cancer cell lines									
BHY	Native +			++		+			(94)
HN	Native ++			+		++			(94)
Human samples									
Normal			Normal > cancer						(95)
Cancer									

mRNA: messenger RNA; pG: pre-proghrelin; native: naturally occurring mature ghrelin; daG, des-acylated ghrelin; aG, acylated ghrelin; GHS-R, ghrelin hormone receptor; In-1: intron-1 ghrelin; ex 3-del: exon 3-deleted ghrelin; GOAT: Ghrelin-O-Acyltransferase; GHROS LncRNA: ghrelin opposite strand long non-coding RNA; EGJA: esophago-gastric junctional adenocarcinoma; SCC: squamous cell carcinoma; SCLC: small cell lung cancer; NSCLC: non-small cell lung cancer; CADS: cancer-associated dyspepsia syndrome.

*+: positive expression; –: negative expression; –/+: equivocal expression; number of + indicate relative expression*.

* *type of ghrelin receptor not specified in corresponding reference*.

Number in parenthesis indicate number of references supporting corresponding data. Numbers in format of x/y indicate number of sample size in corresponding reference with positive results.

*Commas separate data of different references in case of each parameter*.

#### Expression of Ghrelin Receptor

GHSR1b expression was detected in breast cancer cell lines (11, 12). However, the majority of *in-vitro* studies did not report GHSR1a expression (12, 29). Similarly, GHSR1b was highly expressed in breast malignant tissues, while it was not present in normal mammary tissue (11, 12). Studies have shown low levels of GHSR1a expression in both normal and malignant breast tissues. However, studies have come to different conclusions with regards to the difference between GHSR1a expression in normal and malignant tissues. One study reported lower GHSR1a expression in cancerous tissue compared to non-cancerous tissue (58); however, the difference was non-significant in another study (12). The differential expression of GHSR1b in normal and malignant breast tissues implies its probable role in breast cancer pathophysiology.

#### Expression of Variant Ghrelin Peptides

Ghrelin gene produces heterogeneous peptide variants. Full-length and exon 3-deleted pre-proghrelin was transcribed both in benign, MCF-10A, and malignant breast cancer cell lines, MDA-MB-231, MCF-7, MDA-MB-435, and T47D. Unlike similar expression levels of full-length pre-proghrelin between benign and malignant cell lines, exon 3-deleted preproghrelin mRNA was significantly more abundant in some malignant cell lines (MDA-MB-231 and MDA-MB-435 but not MCF-7) compared to a benign cell line (11). Similarly, exon 3-deleted preproghrelin showed more intense expression in cancerous compared to normal breast tissues. Staining became more intense in tissue sections of tumors with higher grades (11).

In1-ghrelin variant retains the copy of intron-1 in the expression of ghrelin gene. In1-ghrelin expression was higher in the invasive breast cancer cell-line, MDA-MB-231, compared to non-invasive MCF-7 breast cancer cell line (59). Similarly, expression of In1-ghrelin was 8-fold upregulated in high-grade breast tumor samples relative to normal tissues. Expression of this variant was highly correlated with proliferation and mitotic markers, Ki-67 and cyclin D3. GHSR1a and GHSR1b expressions were significantly correlated with In1-ghrelin level, while none of these receptors correlated with native ghrelin expression (12). In1-ghrelin expression was not linked to ER, PR, and HER2/neu status (positive correlation between HER2/neu and In1-ghrelin expressions was not statistically significant). Augmented expressions of exon 3-deleted ghrelin and In1-ghrelin in breast cancer may indicate the importance of these peptides in cancer pathophysiology.

Some evidence showed opposite regulation of ghrelin and In1-ghrelin. Although ghrelin, des-acylated ghrelin and Tamoxifen upregulated ghrelin expression in the malignant cell line, they reduced In1-ghrelin expression. Because these effects were not seen by estradiol, authors postulated that Tamoxifen affects ghrelin expression by an ER-independent manner (12).

#### Circulating Ghrelin Level

We know little about levels and clinicopathologic roles of circulating ghrelin in breast cancer patients (60, 61, 98). A case-control study reported comparable circulating ghrelin levels in hereditary breast cancer patients (*n* = 25) and their healthy relative controls (*n* = 38) (98). More research on circulating ghrelin in breast cancer is warranted.

#### Expression of Ghrelin Processing Enzyme

High expression of GOAT was detected in MDA-MB-231 cancer cell line (12). Furthermore, it was upregulated in malignant breast tissues. GOAT expression was correlated with the expression of In1-ghrelin variant to support the assumption that In1-ghrelin might be the main substrate for GOAT (12).

#### Exogenous Native and Variant Ghrelin Peptide

Treatment by higher doses of both acylated ghrelin and des-acylated ghrelin inhibited proliferation of MCF-7 cancer cell line (29). The inhibitory effect on MDA-MB-231 was only seen by acylated ghrelin treatment. In contrast, treatment by lower doses of acylated ghrelin enhanced proliferation rates of some breast cell lines, MDA-MB-231 and MDA-MB-435, but not MCF-7 and normal MCF-10A (11, 59). A similar differential pattern was reported for exon 3-deleted pre-proghrelin mRNA among cell lines (11), implying a role for this ghrelin variant in the response of cancer cells to exogenous native ghrelin. Both In1-ghrelin peptide treatment and its transfection into MDA-MB-231 and MCF-7 breast cancer cell-lines increased proliferation, migration, and sphere-formation (12, 59). These effects accompanied upregulation of TGF-β1, JAG1, and β-catenin expression (59). Differences in type and dose of exogenous ghrelin and type of cell line used could explain these different observations.

#### Ghrelin and Ghrelin Receptor Genes Polymorphism

In a nested case-control study (648 cases/659 controls), none of the tagging single-nucleotide polymorphisms (SNP) in the *GHRL* gene were associated with the risk of breast cancer development (99). However, another nested case-control (1,359 cases/2,389 controls) reported associations of some polymorphisms (*GHRL* rs171407-G allele and *GHSR* 2948694-GG genotype) with increased risk of breast cancer (100). Finally, associations of Gly57Gly SNP of *GHSR* with increased risk, and association of some rare haplotypes of *GHRL* with reduced risk of breast cancer were reported. Authors did not find altered risk with individual SNPs of *GHRL* (101). Differences in distribution of breast cancer subtypes, sample size, ethnicity, and age of the study populations could explain controversial results.

### Attribution of Ghrelin Axis to Summary Clinical Survival

#### Expression of Ghrelin Peptides and Receptor

Positive ghrelin immune-reactivity of tumor tissue associated with better survival, independently of other putative prognostic factors such as grade and stage of the tumor (Table 2; Supplementary Table 1) (96). Likewise, ghrelin expression was associated with favorable breast cancer-specific survival in male breast cancer patients, irrespective of primary tumor size and lymph node involvement. Ghrelin status did not affect response to adjuvant endocrine therapy among ER-positive cases (97). Further, ghrelin positivity of tumor cells was associated with a decreased mortality when comparing 190 patients who died of breast cancer and 190 breast cancer patients as living controls (127). These findings are in line with findings of inverse associations between native ghrelin expression and proliferation, size, and grade of breast tumor cells (96). In contrast, higher expressions of In1-ghrelin associated with shorter disease-free survival in 117 cases of grade three breast cancer (59).

**Table 2:** Summary of *in-vitro, in-vivo*, and clinical data concerning the role of ghrelin-axis in development and progression of different types of cancer.

**Breast cancer**				**Gastric cancer**			
***In-vitro***			**Clinical**	***In-vitro***		***In-vivo***	**Clinical**
**MDA-MB231**	**MCF-7**	**MB-435**	**Carcinoma**	**AGS**	**GES-1**	**SGC7901**	**Adenocarcinoma**
**Cell viability** *Proliferation:* By aG: ↓, By In-1: , By ghrelin: *Stem cell activity:* By ghrelin: ↔ By In-1: **Cell invasion/migration** By ghrelin: ↔ By In-1:	**Cell viability** *Proliferation:* By aG: ↓, ↔ By daG: ↓ By ghrelin: ↔ By In-1: *Stem cell activity:* By ghrelin: ↔ By In-1: **Cell invasion/migration** By ghrelin: ↔ By In-1:	**Cell viability** *Proliferation:* By aG:	**Cancer development/progression***Risk:* *Ghrl* SNPs ↔ (2 ) *Ghrl* haplotypes GGAC and GGAT ↓ *Ghsr* SNP Gly57Gly *Ghrl* rs171407-G allele *Ghsr* rs2948694-GG genotype Plasma ghrelin ↔*Progression:* Lymph node involvement: Tissue *GHS-R1a* peptide ↓, In-1 gene expression Grade: Tissue ex 3-del ghrelin peptide Survival: Tissue ghrelin peptide level (3 ), *Ghrl* rs27647-GG SNP ↓, *Ghrl* rs3755777-C SNP , In-1 gene expression ↓	**Cell viability** *Proliferation:* By ghrelin: By daG: **Cell invasion/migration** By ghrelin:	**Cell viability***Apoptosis:* By ghrelin: ↓**Cell invasion/migration** By ghrelin:	**Cancer development/ progression** *Development:* Tumor size: xenograft ghrelin expression	**Cancer development/ progression** *Risk:* Serum total ghrelin ↓*Progression:* Grade: plasma total ghrelin , ↓ Survival: pre-operative plasma ghrelin and aG biphasic, post-operative plasma ghrelin
(11, 12, 29, 59)	(11, 29, 59)	(11)	(11, 96–103)	(37, 47)	(37, 104)	(37)	(65, 67) (50) (105)
**Oesophageal cancer**					**Pancrease cancer**					
***In-vitro***	**Clinical**				***In-vitro***				***In-vivo***	**Clinical**
**OE-19**	**Adenocarcinoma**	**EGJA**	**SCC**	**Carcinoma (general)**	**PANC1**	**MIAPaCa2**	**BxPC3**	**Capan2**	**MIAPaCa2**	**Neuroendocrine tumor**
**Cell viability** *Apoptosis:* By ghrelin: ↔ **Anti-inflammatory effect** *TNFα-induced COX-2*: By ghrelin ↓ *IL-1β expression*: By ghrelin ↓	**Cancer development/progression***Risk:* Serum total ghrelin ↓, ↔ *Ghrl* SNPs↔	**Cancer development/progression***Risk:* *Ghrl* SNPs ↔ Serum total ghrelin ↓	**Anti-inflammatory effect** *SIRS durations*: ghrelin IV infusion ↓ *CRP*: ghrelin IV infusion ↓ **Cancer development/progression***Risk:* *Ghrl* SNPs↔ Serum total ghrelin ↓ (2)*Progression:* Stage: tumor tissue ghrelin (2) Invasion: tumor tissue ghrelin Grade: tumor tissue ghrelin ↓ Venous invasion: tumor tissue ghrelin Survival: tumor tissue ghrelin↔	**Cancer development/progression** *Progression:* Clinicopathologic features: Serum total ghrelin ↔ Weight loss: post-operativeghrelin ↔	**Cell viability** *Proliferation:* By ghrelin: PANC1 >MIAPaCa2 > BxPC3 > Capan2 **Cell invasion/migration** By ghrelin: PANC1 >MIAPaCa2 > BxPC3 > Capan2				**Cancer development/progression** *Development:* Tumor size: ghrelin treatment ↓*Progression:* Body weight: ghrelin treatment↔	**Cancer development/progression** *Progression:* Survival: tumor tissue ghrelin ↔, aG/total ghrelin ratio ↔ Anorexia: aG/total ghrelin ratio ↓
(69)	(48, 71, 106)	(49, 106)	(43, 48, 72, 106, 107)	(45, 108)	(38)				(109)	(84 , 110 )
**Colorectal cancer**							
***In-vitro***				***In-vivo***			**Clinical**
**HT-29**	**HCT-15**	**SW-48**	**RKO**	**AOM-DSS**	**APC mutant**	**Colon 26 tumor**	**Carcinoma**
**Cell viability** *Proliferation:* By ghrelin:	**Cell viability***Proliferation:* By ghrelin:	**Cell viability** *Proliferation:* By *GHS-R* antagonist: ↓ By anti-ghrelin antibody: ↓	**Cell viability***Proliferation:* By *GHS-R* antagonist: ↓ By anti-ghrelin antibody: ↓	**Anti-inflammatory effect** *Gene expression:* IL-1β: aG ↓ IL-6, TNF-α, IFN-γ :aG ↔ *Peptide level* F4/80 and MPO: aG ↓		**Cancer development/progression***Development:* Tumor weight: *GHS-R* agonist ↔ (2 )	**Cancer development/progression***Risk:* *Ghrl* rs27647-T allele ↓, *Ghrl* rs35683-C allele ↓ (Czech) *Ghrl* rs27647-T allele ↔, *Ghrl* rs35683-C allele ↔ (Germany) Serum total ghrelin: overall risk↓, risk in <5 years of blood draw ↔, risk in <10 years of blood draw ↓, risk in 10–20 years of blood draw ↔ (2), risk in >20 years of blood draw *Progression:* Stage: serum total ghrelin↓, plasma ghrelin , tumor tissue ghrelin , tumor tissue GHS-R1a ↓, tumor tissue GHS-R1b Tumor size: plasma ghrelin Tumor Grade: plasma ghrelin Survival: plasma ghrelin↔, *GHRLOS* LncRNA↓ Lymph node involvement: *GHRLOS* LncRNA↓ Distant metastasis: *GHRLOS* LncRNA↓
		**Cell invasion/migration** By *GHS-R* antagonist: ↓ By anti-ghrelin antibody**:** **↓**	**Cell invasion/Migration** By *GHS-R* antagonist: ↓ By anti ghrelin Antibody: ↓	**Cancer development/progression** *Development:* Tumor number: aG ↓, *Ghrl* deletion ↔	**Cancer development/progression** *Development:* Tumor number: aG ↔, *Ghrl* deletion ↔		
(34)	(34)	(35)	(35)	(42)	(42)	(111) (112)	(35 , 46 , 62 , 64 , 113 –115 )
**Lung cancer**								
***In-vitro***					***In-vivo***		**Clinical**	
**CALU-1 (Non-endocrine)**	**HLC-1 (Non-endocrine)**	**A549 (Adenocarcinoma)**	**H1650 and HCC827/GR (Drug-resistant NSCLC)**	**H345 (Neuroendocrine)**	**Urethane-induced adenocarcinoma**	**Lewis carcinoma**	**NSCLC**	**SCC**
**Cell viability***Proliferation:* By ghrelin: ↔	**Cell viability** *Proliferation and Viability:* By aG: ↔*Apoptosis* By aG: ↔	**Cell viability** *Proliferation:* By aG:	**Cell viability***Proliferation:* By GHSR antagonist: ↓*Apoptosis:* By GHSR antagonist:	**Cell viability***Proliferation:* By aG: ↓ By daG: ↓*Apoptosis:* By aG:	**Cancer development/progression***Progression:* Animal weight: aG	**Cancer development/progression** *Progression*: Animal weight: aG Tumor weight: aG ↔ Survival: aG	**Cancer development/progression***Progression:* Survival: plasma Ghrelin ↔ (2) GHS-R agonist ↔ (2) Lean body mass: GHS-R agonist (2), ↔ Body weight: GHS-R agonist (2) Appetite: GHS-R agonist Handgrip strength: GHS-R agonist ↔ (3) Fatigue: GHS-R agonist ↔	**Cancer development/progression***Progression:* Survival: plasma Ghrelin ↔
(73)	(116)	(117)	(74)	(75)	(118)	(119)	(77) (44) (120) (121) (122) (123)	(44)
**Prostate cancer**							
***In-vitro***						***In-vivo***	**Clinical**
**DU145**	**LNCaP**	**PC3**	**22Rv1**	**VCaP**	**Normal cell line**	**PC3**	**Carcinoma**
**Cell viability** *Proliferation:* By aG: ↓, ↔ By daG: ↓ By In-1:	**Cell viability** *Proliferation:* By aG: ↔ (2), By daG: ↔ By ex 3-del: ↔ By In-1:	**Cell viability** *Proliferation:* By ghrelin: By GHSR antagonist: ↔ By aG: biphasic, (2) By daG: biphasic By ex 3-del: ↔ By In-1:	**Cell viability***Proliferation:* By aG: By In-1:	**Cell viability** *Proliferation:* By aG: ↔ By In-1:	**Cell viability***Proliferation:* By aG: ↔ By In-1: ↔	**Cancer development/progression** *Development:* Tumor volume: daG *↔*, GHSR antagonist ↓ In-1 transfection > aG transfection Tumor weight: GHSR antagonist ↓ Tumor Ki67: daG *↔*, GHSR antagonist ↔ Tumor CD31: daG: ↔ Tumor EGFR: GHSR antagonist ↓	**Cancer development/progression***Progression:* Gleason score: plasma GOAT Metastasis: plasma GOAT Progression markers: In-1 gene expression , ghrelin gene expression ↔
(78, 80)	(36, 78, 80)	(36, 79, 80, 124)	(78)	(78)	(78)	(78, 124, 125)	(78, 83)
**Clear cell subtype of renal cell carcinoma**						**Oral squamous cell carcinoma**		
***In-vitro***				***In-vivo***	**Clinical**	***In-vitro***		**Clinical**
**786-0**	**ACHN**	**A-498**	**Cancer cell-line panel**	**786-0**	**Carcinoma**	**BHY**	**HN**	**Cancer**
**Cell invasion/migration** By ghrelin: ACHN > A-489 >786-0			**Cell invasion/migration** By ghrelin Overexpression:	**Cancer development/progression***Progression:* Number of metastatic lung nodule: ghrelin gene knocked out mice < ghrelin naïve mice	**Cancer development/progression***Progression:* Tumor progression: ghrelin Survival: ghrelin ↓ (2)	**Cell viability** *Proliferation:* By aG: HN , BHY HN > BHY		**Cancer development/progression***Progression* Grade: tumor ghrelin ↓
(85)	(126)	(85)	(85)	(94)	(95)
**Central nervous system cancer**					
***In-vitro***		**Clinical**			
**Astrocytoma (CCF-STTG1, U-87, U-118, SW1088)**	**Glioma: C6 (rat), U251(human)**	**Glioblastoma**	**Anaplastic astrocytoma**	**Diffuse astrocytoma**	**Neurofibromatosis**
**Cell viability** By aG: SW1088 > U87 > CCF-STTG1 > U118	**Cell viability** By ghrelin: U251 > C6	**Cancer development/progression** *Progression:* Tumor cell proliferation: Ghrelin/GHS-R1a (in anaplastic astrocytoma and glioblastomas) Survival: ghrelin/GHSR-1a ratio ↓			**Cancer development/progression***Progression:* Tumor size: tumor GHSR expression
(86)	(87)	(88)			(68)
**Endometrial cancer**			**Ovarian cancer**
***In-vitro***	***In-vivo***	**Clinical**	***In-vitro***
**Ishikawa, KLE (intact and GHSR-1a KO)**	**Ishikawa (intact and GHSR-1a KO)**	**Cancer**	**HO-8910**
**Cell viability** *Proliferation:* with/without ghrelin: GHSR-1a KO < intact	**Cancer development/progression***Development:* Tumor size: GHSR-1a KO < GHSR-1a intact*Progression:* Tumor Ki67: GHSR-1a KO < GHSR-1a intact	**Cancer development/progression** *Progression:* Grade: tumor ghrelin ↔, tumor GHSR1a ↔, tumor total ghrelin↓ Stage: tumor total ghrelin ↔ Lymph vascular invasion: tumor total ghrelin ↔	**Cell viability***Proliferation:* By ghrelin: ↓
(92)	(92)	(92, 93)	(90)

*daG: des-acylated ghrelin; aG: acylated ghrelin; GHS-R: ghrelin hormone receptor; In-1: intron-1 ghrelin; SNP: single nucleotide polymorphism; ex 3-del: exon 3-deleted ghrelin; GOAT: Ghrelin-O-Acyltransferase; EGJA: esophageo-gastric junctional adenocarcinoma; SCC: squamous cell carcinoma; SIRS: systemic inflammatory response syndrome; CRP: C-reactive protein*.

*(increased/improved/positive association); ↓(decreased/deteriorated/negative association); ↔(no effect/association)*.

*Number in parenthesis indicate number of references supporting corresponding data*.

*Commas separate data of different references in case of each parameter*.

#### Ghrelin and Ghrelin Receptor Genes Polymorphism

Polymorphism of *GHRL* gene was linked to both all-cause and breast cancer-specific mortalities. *GHRL* rs27647-GG genotype was correlated with worse all-cause mortality, whereas *GHRL* rs3755777-C allele conferred overall survival advantage (102). Future studies can explore if these polymorphisms alter expression of ghrelin peptide and receptors.

## Colorectal cancer

### Attribution of Ghrelin Axis to Cancer Pathogenesis and Differentiation

#### Expression of Native Ghrelin Peptide

Both normal and malignant human colon cell lines express ghrelin (Table 1; Supplementary Table 2). Expression of ghrelin is upregulated in malignant compared to normal cell lines and in cancerous compared to adjacent normal tissue samples taken from cancer patients. Immune-reactivity to ghrelin was stronger in samples from higher stage tumors. A similar correlation was observed between ghrelin expression and tumor grade. However, poorly differentiated tumors demonstrated dramatic reductions in immune-reactivity to ghrelin. It potentially rules out a limiting dependency of cancer cells on tumor-produced ghrelin as they still survive even when ghrelin expression is lost in high grade tumors (35).

#### Expression of Ghrelin Receptor

GHSR1a and GHSR1b were present in both normal and malignant cell lines. However, malignant cell lines expressed higher GHSR1b. With the increasing stage of cancer patients, tumor expression of GHSR1a and GHSR1b decreased and increased, respectively (35). Alterations of ghrelin receptor expressions are in line with breast cancer, implying a more important role of GHSR1b in cancer biology.

#### Circulating Ghrelin Level

In a nested case-control (523 cases/523 controls) of male smokers, colorectal cancer incidence was higher in subjects within the lowest quartile compared to those within the highest quartile of serum total ghrelin before cancer occurrence. This was the case for patients with their cancer diagnosed within 10 years of blood draw; however, patients with diagnosed after 20 years of blood draw showed a positive association between serum total ghrelin and colorectal cancer risk (46). No association was reported for the 10–20-year interval group. Another nested case-control (60 cases/60 controls) did not show an association between cancer risk and blood ghrelin level sampled ≤ 5 or 10–20 years before cancer diagnosis. Furthermore, there was not significant within-individual changes in ghrelin level during the time considered (113).

Two studies reported lower levels of ghrelin in colorectal cancer patients than healthy control subjects (62, 63). Serum ghrelin level showed an inverse association with tumor stage and was higher in patients with anti-Helicobacter pylori Ab. Despite inverse association of ghrelin with BMI in healthy controls, no such correlation was reported in cancer patients (62). Conversely, another study reported higher serum total ghrelin in cancer patients (patients with BMI more than 30 and <16 were excluded). Ghrelin level was higher in patients with larger tumor size, higher stage, and higher grade. No association between ghrelin and patients‘ BMI was reported (64). Waseem et al. showed that serum ghrelin was higher in cachectic cancer patients compared to non-cachectic patients, which could be a compensatory response to devastating metabolic conditions. Additionally, ghrelin levels were not correlated with stage, grade, and BMI (35). Different BMI distribution of cases includes in each study might explain some of controversy.

#### Long Non-coding Ribonucleic Acids (LncRNA)

The LncRNA *GHRLOS* is transcribed from the antisense strand of ghrelin gene; however, it is not translated (128). Compared to adjacent normal tissue, 54.4% (199/366 cancer patients) of tumor specimens showed reduced *GHRLOS* transcript. Downregulation of *GHRLOS* was associated with more aggressive tumor phenotype (114).

#### Exogenous Native Ghrelin

Ghrelin promoted cancer cell proliferation. The pro-proliferative effect of ghrelin was attenuated by GHSR inhibitor ([D-Lys3]-GHRP-6), dominant-negative mutation of Ras, PI3K inhibitor (LY 294002), dominant-negative mutation of Akt, or mTOR inhibitor (rapamycin). These findings imply the functional role of Ras/PI3K/Akt/mTOR pathway in ghrelin-induced cancer cell proliferation (34).

In a mouse model of inflammation-associated colorectal carcinogenesis induced by Azoxymethane/Dextran Sodium Sulfate (AOM/DSS), exogenous ghrelin treatment reduced tumor burden and increased body weight. Ghrelin administration resulted in reduced infiltration of macrophages and neutrophils in ulcerated colon tissue. In contrast, Ghrelin treatment did not alter tumor incidence or size in APC-mutant mice (42) or colon-26 tumor bearing mice (111, 112).

#### Exogenous Ghrelin Inhibitors

Either GHSR antagonist or neutralizing anti-ghrelin Ab diminished proliferation and invasion of malignant cell lines (35). In the AOM/DSS model, the number of tumors was not different between *Ghrl*-deleted mice and naïve mice. However, *Ghrl*-deleted mice had larger tumors. The proliferation, apoptosis, and angiogenesis indices did not show a difference between groups. In APC-mutant mice, the deletion of ghrelin gene did not affect the size or number of tumors (42). These findings suggest tumor-suppression by anti-inflammatory effects of systemic ghrelin, which is more pronounced in exogenous ghrelin.

#### Ghrelin and Ghrelin Receptor Genes Polymorphism

*GHRL* rs27647-T and rs35683-C alleles were associated with decreased risk of colorectal cancer in Czech Republic (680 cases/593 controls), but not German (569 cases/726 controls), population (115).

### Attribution of Ghrelin Axis to Summary Clinical Survival

After adjusting for its association with tumor stage, serum total ghrelin was not correlated with survival (Table 2; Supplementary Table 2) (64). Low levels of LncRNA *GHRLOS* in tumor samples of cancer patients were associated with a shorter survival (114). Evaluation of prognostic roles of pre- and post-surgical circulating ghrelin can provide clues about specific roles of local and systemic ghrelin in colorectal cancer (64).

### Implications of Ghrelin Axis in Cancer Therapy

GHSR agonists, HM01 and Z505 Hydrochloride, showed beneficial effects on cachexia in mouse models of colon cancer. However, the treatment did not alter tumor weight (84, 106).

## Gastric cancer

### Attribution of Ghrelin Axis to Cancer Pathogenesis and Differentiation

#### Expression of Native Ghrelin Peptide

Ghrelin was not expressed in the AGS cell line (Table 1; Supplementary Table 3) (47). Compared to normal tissue, tumor tissue expressed negligible amount of ghrelin (65, 66). The low level of ghrelin in tumor tissue suggests impairment of ghrelin production in damaged gastric mucosa. However, there was no association of local ghrelin expression with the extension and location of the tumor (65).

#### Expression of Ghrelin Receptor

Ghrelin receptor is up-regulated in malignant cell lines (37, 47) and tumor tissue (66). AGS cells expressed both GHS-R1a and GHS-R1b (47) and GHS-R expression was higher in SGC7901 compared to AGS (37).

#### Circulating Ghrelin Level

Plasma ghrelin was lowest in 23 GC patients compared to patients with benign conditions, such as acute gastritis or gastric ulcer (67). Likewise, GC patients associated with H. pylori infection had low ghrelin levels. Authors suggested plasma ghrelin in GC is influenced by the extent of the tumor and H. pylori-induced atrophic gastritis. Plasma ghrelin drops significantly after total gastrectomy (50, 65), showing the stomach as the main source of ghrelin. Blood ghrelin recovered to some extent thereafter, suggesting compensatory production of ghrelin from other non-gastric organs (65). No difference was observed in serum ghrelin between healthy cases and patients with gastric neuroendocrine tumor (68).

In a nested case–control (261 non-cardia GC/441 control) (49), risk of non-cardia GC was higher for those in the lowest quartile of serum ghrelin compared vs. highest quartile. The association could be due to atrophic gastritis as a reason for both lower ghrelin and higher risk of cancer. Authors also considered anti-inflammatory effects of ghrelin as another possible mechanism. Likewise, inverse association of serum total ghrelin with risk of non-cardia and cardia GC adjusted for potential confounders was reported (48). Potential use of ghrelin in identifying early GC can be explored.

Patients with undifferentiated adenocarcinoma had higher plasma ghrelin compared to those with differentiated tumor (67). Similarly, undifferentiated tumor tissue expressed higher ghrelin than differentiated tumor tissue (65). No association was reported between ghrelin and other clinicopathological factors (e.g., tumor stage).

#### Exogenous Native Ghrelin Peptides

Both ghrelin and des-acylated ghrelin induced proliferation of adenocarcinoma AGS cells via activation of the ERK1/2 and PI3K/Akt pathway (47, 104). Different concentrations of ghrelin and des-acylated ghrelin could increase cells in the S phase of the cell cycle. Furthermore, ghrelin could promote proliferation, migration, and invasion of AGS and SGC7901 cells via GHS-R/NF-jB signaling pathway (37). Ghrelin promoted CDK6 expression, suppressed P53 expression, and upregulated the metastasis factor MMP2 expression. Likewise, ghrelin administration increased tumor size in mice.

### Attribution of Ghrelin Axis to Summary Clinical Survival

#### Circulating Ghrelin Level

Higher post-operative plasma ghrelin was linked with better prognosis in GC patients (Table 2; Supplementary Table 3) (50, 105). This survival advantage was independent of stage, grade, cachexia, and tumor size or location. However, the pre-operative plasma ghrelin showed a different prognostic pattern (50). Patients with either the lowest or highest quartile of plasma ghrelin showed higher survival rates than patients with second-third quartiles. The difference in prognostic patterns of pre-operative and post-operative plasma ghrelin supports the interaction between opposite local and systemic effects of ghrelin in determining the final summary effect. The unfavorable paracrine effects of ghrelin play some role when tumor cells are still exposed to local ghrelin before gastrectomy. However, the prognostic pattern of post-operative blood ghrelin, wherein tumor cells are no longer exposed to local ghrelin, was purely dominated by protective systemic effect. Therefore, ghrelin could be considered a promising prognostic biomarker and therapeutic option in GC like other novel candidates introduced (129, 130).

## Oesophageal Cancer

### Attribution of Ghrelin Axis to Cancer Pathogenesis and Differentiation

#### Expression of Native Ghrelin Peptide

Ghrelin expression was negligible in tumor specimens of esophageal adenocarcinoma (OA) (70). However, high ghrelin expression was observed in 29.0% of patients with squamous cell carcinoma (OSCC) (107) that was associated with depth of tumor invasion and grade (Tables 1, 2; Supplementary Table 4).

#### Expression of Ghrelin Receptor

GHSR1a gene expression was higher in biopsies from Barrett's esophagus compared to normal squamous epithelium tissue (69).

#### Circulating Ghrelin Level

Individuals with low serum ghrelin had 5-fold and 7-fold higher risks of esophagogastric junctional adenocarcinoma (OGJA) and OSCC, respectively (49, 72). Authors attributed this association to the presence of atrophic gastritis. A population-based study on OSCC cancer reported similar findings (48). The association was independent of atrophic gastritis markers. Therefore, they argued the role of atrophic gastritis in ghrelin-cancer risk association. A strong inverse association between serum ghrelin and risk of OA cancer was also reported (71). This association was restricted to overweight patients. Authors contributed this inverse association to anti-inflammatory effects of ghrelin. In contrast, a case-control reported no association between serum ghrelin and risk of OA (48).

Post-operative plasma ghrelin was lower than the pre-operative concentrations in esophageal cancer patients, which slightly recovered in 6–24 months. There was no relationship between circulating ghrelin and clinicopathologic features of tumor (45, 108).

#### Exogenous Native Ghrelin Peptides

Treatment of OE-19 cell line with different concentrations of ghrelin revealed an inhibitory effect on Barrett's carcinogenesis via anti-inflammatory mechanism (69). Ghrelin attenuated the level of TNFa-induced COX-2 and IL-1b expression. Likewise, exogenous ghrelin was safe and reduced post-operative systemic inflammation (duration and intensity) in esophageal cancer patients (43).

#### Ghrelin and Ghrelin Receptor Genes Polymorphism

An Australian case-control (260 OA/301 OGJA/213 OSCC cases/1,352 controls) revealed no major association between ghrelin gene SNPs and cancer incidence. A modest positive association between rs696217 SNP and OA cancer was reported in cases with BMI < 25 kg/m^2^ (70).

### Attribution of Ghrelin Axis to Summary Clinical Survival

#### Expression of Native Ghrelin Peptide

A trend of worse 1-year survival was reported in cancer patients positive vs. negative for local tumor expression of ghrelin (Table 2; Supplementary Table 4) (107). This finding was in line with the association of local ghrelin with tumor invasion. The 5-year survival rates showed no difference. Because local and systemic ghrelin exert opposite effects in esophageal cancer, investigating the prognostic role of circulating ghrelin will be more elucidative.

## Lung Cancer

### Attribution of Ghrelin Axis to Cancer Pathogenesis and Differentiation

#### Expression of Native Ghrelin Peptide and Its Receptor

Expression of ghrelin and its receptors seems to be linked to the histological subtype of lung cancer (Table 2; Supplementary Table 5). Ghrelin and GHSR1a are more abundantly expressed in tumors of neuroendocrine origins compared to those of non-endocrine origins (73, 75). Only one study reported expression of GHSR1b in non-small cell lung carcinoma (131). To our knowledge, expression of GHSR1b has not been investigated in other subtypes of lung cancer. Furthermore, GHSR expression was linked to the activation of the MAPK/ERK and PI3K/AKT signaling and accompanied cell proliferation in Gefitinib (epidermal growth factor tyrosine kinase inhibitor)-resistant non-small cell lung cancer cell lines (74).

#### Circulating Ghrelin Level

Alterations of serum ghrelin in patients with lung cancer is most probably related to tumor-driven systemic catabolic and inflammatory state. Two studies reported higher levels of circulating ghrelin in cancer patients compared to healthy controls (44, 77). Ghrelin level was not associated with stage and histological subtype of tumor. The association of ghrelin with body weight loss (BWL) was conflicting. This could have been due to different definitions of BWL in two studies. The study that reported positive association of ghrelin with BWL used higher cut-off (10 vs. 5%) for weight loss definition (77). Ghrelin level was even higher in cancer patients with BWL compared to cancer patients without BWL (77). Another study on a smaller sample size reported comparable plasma levels of ghrelin between cancer patients and healthy controls. Yet, patients with cachexia had higher levels of plasma ghrelin compared to non-cachectic patients (76).

#### Exogenous Native Ghrelin

Ghrelin had differential effects on lung cancer cell lines, some of which could be explained by their origin (endocrine vs. non-endocrine) and subtype. Ghrelin treatment did not affect cell proliferation in CALU-1 cancer cell line (non-endocrine origin) (73). Similarly, *in-vitro* administration of ghrelin did not significantly change viability, proliferation, apoptosis, and cellular respiration in HLC-1 lung adenocarcinoma cell line (non-endocrine) (99). In contrast, enhanced proliferation of A549 lung adenocarcinoma cell line upon ghrelin treatment was recently reported. The ghrelin effect on proliferation was mediated by GHSR and at least two independent intracellular signaling pathways, including PI3K/Akt/mTOR/P70S6K and ERK molecules, were involved in the process (117). Finally, GHSR antagonist attenuated cell proliferation associated with MAPK/ERK and PI3K/AKT signaling in Gefitinib-resistant non-small cell lung cancer cell lines (74).

Both ghrelin and des-acylated ghrelin inhibited proliferation of H345 small cell lung carcinoma cell line (neuro-endocrine). GHSR1a and GHSR1b mRNA were not detected by RT-PCR in this cell-line. Absence of expression of known ghrelin receptors may indicate existence of other modes of action for ghrelin. Co-administration of ghrelin with a tyrosine phosphatase inhibitor abolished ghrelin anti-proliferative effects on H345 cells, indicating that dephosphorylation could be a crucial step in the signaling pathway(s) underlying inhibitory effects of ghrelin on this cell line. Ghrelin activated apoptosis pathway could also explain growth-modulating effects of ghrelin on H345 cell-line (75).

### Attribution of Ghrelin Axis to Summary Clinical Survival

Two studies reported no associations between ghrelin level and overall survival in cancer patients (44, 77). Ghrelin was not associated with tumor response to therapy, either (44).

### Implications of Ghrelin Axis in Cancer Therapy

Ghrelin administration alleviated tumor-induced cachectic conditions in mouse models of Lewis lung carcinoma and adenocarcinoma (118, 119). Although ghrelin treatment did not change tumor mass, it improved survivals of Lewis lung carcinoma mice (119). This survival advantage could be due to an improvement in cachexia, but doesn't have a direct effect on tumor.

Anamorelin, a ghrelin receptor agonist, has been studied for the treatment of cachexia in patients with non-small cell lung cancer (120–123). Phase 3 clinical trials ROMANA 1 and 2 (overall 977 patients) showed that the administration of Anamorelin was effective in increasing body weight in cancer patients with cachexia. However, it did not alter median survival over 1 year (120).

In conclusion, ghrelin alteration in patients with lung cancer seems to be a secondary systemic response to cancer-induced cachexia (Table 2). Current evidence does not support a link between ghrelin and survival of lung cancer patients. Derived from both *in-vitro* and clinical studies, direct effects of ghrelin on lung carcinoma may be varied based on subtype of tumor. Hence, it is suggested for future studies to evaluate segregated contributions of ghrelin to pathogenesis and prognosis of lung cancer patients based on the origin of their tumors (non-endocrine vs. neuroendocrine) and histologic subtype.

## Other Cancers

### Prostate Cancer

Normal prostate cell line expresses In1-ghrelin and GHSR1-a. GHSR1-b is not expressed and the expression of ghrelin is deemed controversial (78, 79). Cancer cell lines consistently express acylated ghrelin, In1-ghrelin, and exon 3-deleted ghrelin. However, the expressions of ghrelin, GHSR1-a, and GHSR1-b are controversial (36, 78–80) (Tables 1, 2; Supplementary Table 6). Although acylated ghrelin treatment stimulated PC3 and LNCaP proliferation via MAPK/ERK1&2 pathway at the concentration of 10 nM, this effect was not observed for treatment by exon 3-deleted (36). Treatment with In1-ghrelin consistently increases proliferation of both the normal and the cancer cell lines studied. Other studies reported controversial results for ghrelin and ghrelin receptor expression as well as effects of exogenous ghrelin or acylated ghrelin on cancer cell proliferation (36, 78–80). Some of these contradictions can be explained by the different type of cell line studied and the different dose of exogenous ghrelin used. In the study that investigated endogenous expression and exogenous effects of both native and In1 variant ghrelins in different prostate cancer cell lines, tumor xenograft, and human tumor samples, In1-ghrelin showed overall more impactful tumor-promoting and oncogenic effects (78).

Although administration of des-acylated ghrelin did not affect tumor volume in mice with PC3 cell xenograft (125), GHSR antagonist [D-Lys 3]-GHRP-6 reduced the tumor volume (124). Furthermore, mice with In1-ghrelin transfected PC3 xenograft presented larger tumor size compared to mice with acylated ghrelin transfected PC3 xenograft (78).

Serum levels of acylated ghrelin was comparable (78) or higher in prostate cancer patients, compared to benign prostate hyperplasia cases (81). No significant differences in serum levels of total ghrelin have been reported between cancer and hyperplasia/normal cases. However, In1-ghrelin and GOAT concentrations are higher in cancer patients compared to normal cases (78, 80–82). Although ghrelin splice variants are locally expressed in cancer tissue, ghrelin receptor is not expressed and the results for the expression of native ghrelin are controversial. Tumor ghrelin gene expression did not show any association with tumor progression markers (78); however, plasma GOAT concentration and tumor In1-ghrelin gene expression were associated with more aggressive tumor phenotypes (78, 83). In1-ghrelin and GOAT, but not native ghrelin, show a consistent unfavorable impact on prostate cancer aggressiveness and the summary clinical role is dominated by local effects. A recent review emphasized the role of G-coupled ghrelin receptor in prostate cancer (132).

### Pancreas Cancer

GHSR1a and GHSR1b transcripts and proteins are detectable in pancreatic adenocarcinoma cell-lines. The highest level of GHSR1b transcription was seen in PANC1 cell-line. Ghrelin transcript was slightly expressed in the PANC1 cell-line and absent in other cell-lines (Tables 1, 2 and Supplementary Table 6). Exogenous ghrelin at 1–10 nM dose increased proliferation, invasiveness, motility, and Akt phosphorylation of all cell-lines. These effects were more pronounced in PANC1 cell-line. Proliferation was suppressed in 100 nM dose (38). Likewise, a clinical study on patients with pancreatic neuroendocrine tumors reported local co-expression of ghrelin and GHSR in cancer tissue resulted in worse survival (84).

However, administration of systemic ghrelin (30 nM/kg) to a mouse model transplanted with pancreatic carcinoma cell line (BALB/c-nu/nu mice injected subcutaneously with MIA-PaCa2 cell line) led to decreased tumor size compared to untreated control mouse. Although ghrelin inhibited plasma leptin levels, it had no effect on body weight (109). It is debated whether the inhibitory effect observed was due to the dose of ghrelin administrated being in the inhibitory range, or due to the dominancy of systemic effects of ghrelin over local stimulatory effects. Dose escalation of exogenous ghrelin administered could have helped clarify it. In a potential second study, this could be an example of controversy between local and short-term systemic effects of ghrelin on a type of cancer. The summary long-term clinical role of systemic ghrelin should be a trade-off between these two contrary effects. Ratio of systemic acetylated/total ghrelin showed no significant association with survival of advanced pancreas cancer patients (110).

### Renal Clear Cell Carcinoma (RCC)

A study investigated ghrelin effects on RCC in *in-vitro, in-vivo*, and clinical setting. The authors observed higher levels of ghrelin expression in metastatic RCC and ghrelin-GHS-R axis induced the invasion in cancer cell (RCC, 786–0, ACHN, A-498, 769-P, and A-704 human cell lines). This effect was mediated via GHS-R that induced AKt phosphorylation at Ser473 and Thr308 of PI3K/Akt pathway, which finally led to cell migration at acylated ghrelin concentrations of 180 nM. Via this pathway, ghrelin induced Snail expression with subsequent decreased E-cadherin and accelerated migration. Accordingly, AKt silencing by Akt siRNA and Pi3K inhibitor prevented Snail activity and migration. Furthermore, the authors injected ghrelin knock-out 786-0 cell lines into nude-mice and found that metastatic lung nodule numbers were significantly lower in ghrelin knocked-out mice compared to the control group. In accordance with *in-vitro* and *in-vivo* findings, higher local expression of ghrelin was a significant and independent poor prognostic factor in RCC patients. Higher ghrelin RNA expression was also associated with risk of metastasis (85). Another study by the same group examined other mechanisms underlying the promoting effects of ghrelin on *in-vitro* local invasion and *in-vivo* metastasis of RCC (126). Authors revealed the contribution of ghrelin-GHSR1a-Aurora A-MMP10 signaling pathway to local invasion and metastasis of RCC. Furthermore, by looking at the cancer genome atlas (TCGA) database, the authors found that tumor expression of ghrelin, Aurora A, and MMP-10 were predictors of poor prognosis in RCC patients (Tables 1, 2; Supplementary Table 6).

These two studies are good examples of the dominance of ghrelin local effects on cancer cells revealed in *in-vitro*, in summary clinical role of ghrelin in a type of cancer. It should be considered that local expression of ghrelin in RCC tissue might not necessarily reflect systemic levels of ghrelin in these patients and the prognostic role of systemic ghrelin is yet to be inferred. Nevertheless, it could be expected that the positive general roles of systemic ghrelin (orexigenic and anti-inflammatory) have not been able to overwhelm the notorious local effects in this cancer.

### Brain Tumors

Overall, ghrelin exerts pro-oncogenic local effects on brain tumors. Ghrelin and its receptor are expressed in different types of astrocytoma cell-lines. Dixit et al. evaluated ghrelin and GHSR1a expression in CCF-STTG1, U-87, U-118, and SW1088 cell lines. GHSR expression was higher in these cell lines compared to normal astrocytes. Ghrelin- GHSR binding led to more cell motility. Ghrelin upregulated GHSR expression and subsequently the motility via this receptor. This effect was preventable by GHSR antagonist (86). In glioma, ghrelin induced migration in both C6 rat cell-line and U251 human cell-line at the concentration of 30 nM. Ghrelin increased cell migration via GHSR, CaMK II, AMPK, and NF-kB pathways. NF-kβ binding to DNA and subsequent kβ transcriptional activity was increased (87) (Tables 1, 2; Supplementary Table 6).

The central nervous system expresses ghrelin under normal condition. In a clinical study, ghrelin and GHSR1a expressions were assessed by IHC in patients with glioblastoma, anaplastic astrocytoma, and diffuse astrocytoma. Ghrelin expression was observed in all glioblastoma, 12/13 anaplastic astrocytoma, and 2/11 diffuse astrocytoma patients. A similar pattern was observed for GHSR1a expression. Based on age and ghrelin/GHSR expression status, patients were classified into lower-score, moderate, and high-score categories. Multivariable analysis of overall survival indicated that survival rate was significantly higher in the lower-score group compared to the high-score group. No significant correlations were found between ghrelin and GHS-R1a expression (88). Likewise, tumor GHSR expression was associated with higher tumor size in patients with neurofibromatosis 1 (89). Like RCC, this is another example of dominance of *in-vitro* local findings in the summary clinical role of ghrelin in a type of human cancer. Similar considerations for prognostic roles of local vs. systemic ghrelin should be applied here, too.

### Gynecological Cancers

Ghrelin induced apoptosis in HO-8910 ovarian cancer cell-line via GHSR-mediated ERK1/2 phosphorylation at the concentration of 182 nM (90). Tissue expression levels of ghrelin were different among three groups of patients with benign, borderline, and malignant serous ovarian tumors (91). These findings may imply ghrelin contribution to carcinogenesis of this type of cancer.

Another study examined ghrelin role in endometrial cancer in three sections of *in-vitro, in-vivo*, and human tissue microarray assay. They observed 1,000 nM dose of exogenous acylated ghrelin enhanced cell proliferation in intact cell-lines. This effect was not seen for des-acylated ghrelin. In addition, GSHR1a knock-out cell lines showed reduced proliferation in the presence or absence of exogenous ghrelin, regardless of dose or type (Tables 1, 2; Supplementary Table 6). Accordingly, the increase in tumor size was slighter in NOD/SCID mice injected with GHSR-1a knock-out Ishikawa cell line compared to mice receiving intact Ishikawa cell line. Microarray tissue assay detected ghrelin and GHSR1a expressions in both benign and cancerous tissue specimen (92). These findings may emphasize the pro-oncogenic role of ghrelin in endometrial cancer, mainly mediated by acylated ghrelin-GHSR1a axis.

Another clinical study demonstrated lower tissue expressions of ghrelin in cancerous tissue compared to hyperplastic endometrial tissue. This study did not investigate ghrelin expression in normal tissue. Decreased ghrelin level might be a differential diagnosis marker in distinguishing carcinoma from hyperplasia (93).

### Oral Squamous Cell Carcinoma

Exogenous acylated ghrelin at doses of 100 nM−1 μM enhanced cell proliferation of BHY and HN oral SCC cell-lines. This effect was mediated by GSK-3B/beta-catenin pathway and up-regulation of cyclin-D1 and c-myc (94). Although this effect was more pronounced in HN cell-line, this cell-line had a lower expression of GHSR1a and a higher expression of GHSR1b compared to BHY cell-line (Tables 1, 2; Supplementary Table 6). This may imply contribution of receptors other than GHSR1a to ghrelin local effects on oral SCC cell-lines. Another clinical study reported lower tissue expression of acylated and des-acylated ghrelin in oral SCC specimen compared to adjacent non-malignant tissue. This finding could have diagnostic implications (95).

## Discussion

**Figure 3:**
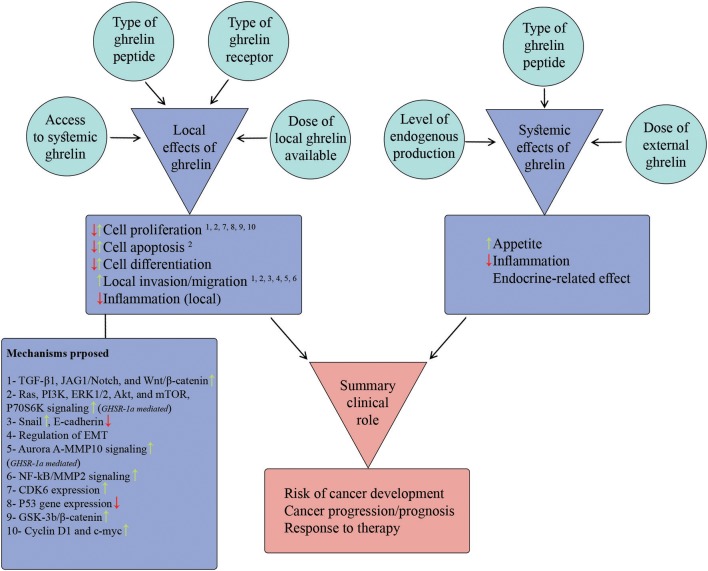
Model proposed to describe summary clinical role of ghrelin in case of each cancer. The figure summarizes the potential local and systemic effects of ghrelin attributable to cancer biology (items listed in two purple boxes) as wells as the candidate factors that regulate these effects (the items inside surrounding circles). Furthermore, the potential mechanisms proposed so far for local effects of ghrelin are listed in the purple box below the list of local effects. The superscript numbers above each local effect correspond to the potential underlying mechanism(s) from the mechanisms list. The summary clinical role of ghrelin is built-up by relative contributions of local and systemic effects. Based on the direction and relative dominance of local and systemic effects, the summary effect on different aspects of cancer (the items in red box) could be promoting or protective.

To address the inconsistencies across studies reporting local effects of ghrelin on different cancer cell-lines, we scrutinized potential factors that regulate/modify cell response to local ghrelin. Some of these factors were inherent to the type of cell-line (e.g., type of ghrelin receptor) and some of them were related to the ghrelin (e.g., type and dose of ghrelin peptide) studied. For controversies between findings of *in-vitro* and *in-vivo*/clinical studies on the same type of cancer, we delineated local and systemic effects of ghrelin on each cancer and discussed the final summary effects observed based on an interaction between local and systemic effects (Figure 3; Table 2). Some effects of ghrelin occur in both local and systemic scales (e.g., anti-inflammatory). Each local and systemic effect of ghrelin could be in favor or against development and progress of corresponding type of cancer.

### Factors Regulating Local Effects

#### Type of Ghrelin Receptor

Type of ghrelin receptor on cell membrane can determine local effects of ghrelin on each tissue/cell-line. Emerging evidence implies that GHSR1a might not be responsible for all ghrelin-mediated effects (25). Des-acylated ghrelin has low binding affinity for this receptor, while recently found to be biologically active (16, 133, 134). Many of the effects attributable to acylated or des-acylated ghrelin could be mediated by other receptors, which are yet to be characterized (25).

Expression of ghrelin receptors is varied among different types of human cancers. Breast and colon cancer cell lines express both GHSR1a and GHSR1b (11, 29, 35). However, ghrelin receptors were not notably expressed in lung cancer cell-lines, specifically in non-endocrine subtype (75, 116). Differential expression of ghrelin receptors was even observed among different cell-lines of the same type of cancer. Unlike other breast cancer cell lines, MDA-MB-231 cell-line expressed only very small amounts of GHSR1a (12). Such a variance also could be expected in expression of unidentified ghrelin receptors. Differential expression of ghrelin receptors would result in varied local effects of ghrelin on tumor cells. Extrinsic ghrelin administration increased proliferation of MDA-MB-231 and MDA-MB-435, but not MCF-7 breast cancer cell lines (11).

Furthermore, despite negligible differences in expression of GHSR1a between normal and cancerous breast tissues, GHSR1b was notably upregulated in cancerous tissue compared to normal tissue (11, 12). Similarly, although normal and malignant human colon cell lines expressed both GHSR1a and GHSR1b, only GHSR1b was upregulated in malignant cells compared to normal tissue (35). Unlike GHSR1a, GHSR1b was positively associated with tumor stage. These observations may imply a more important role of GHSR1b in cancer biology.

Different levels of receptor expression was also seen among tissue samples taken from different stages of a particular cancer (35). With increased stages of disease in colorectal cancer patients, expression of GHSR1a declined whereas expression of GHSR1b increased. This could result in different responses to ghrelin by tumors of different stages. A recent nested case control reported a specific pattern of ghrelin effects on development of colorectal cancer (30). While cancer risk was inversely correlated with serum ghrelin measured within 10 years before diagnosis of cancer, cancer risk increased with higher levels of serum ghrelin measured more than 20 years before diagnosis. In other words, exposure to higher systemic ghrelin resulted in different effects on cancer development depending on the type of pre-cancerous lesion in the time-line of cancer development, exposed. This could be, in part, due to different expression patterns of ghrelin receptors in lesions with different degrees of malignant transformation.

#### Type of Ghrelin Peptide and Its Splice Variants

Each type of ghrelin peptide and its derivatives could have different local effects on cell lines. Acylated and des-acylated ghrelins have similar local effects on human breast (29), lung (75), and gastric cancer (47) cell lines. Seemingly, two natural ghrelins have similar local behavior in most cancer cell-lines investigated. These observations may also imply that two ghrelins use common, still unidentified, receptors in addition to GHSRs, because des-acylated ghrelin has much less affinity to known GHSRs (135). Exceptionally, although acylated ghrelin enhanced cell proliferation in endometrial cancer cell lines, des-acylated ghrelin did not exert proliferative effects (92). Authors observed a significant role of GHSR1a in the cellular growth of endometrial cancer cell-lines, which might explain contradicting results observed for acylated and des-acylated ghrelin.

Native ghrelin showed different local effects on breast cancer cell lines compared to its splice variants, exon 3-deleted preproghrelin and intron-1 retained (Int-1). While the expression of full-length preproghrelin was similar between benign and malignant cell-lines (11), exon 3-deleted preproghrelin mRNA was significantly upregulated in malignant breast tissue and was associated with tumor grade (11). Upregulation of exon 3-deleted preproghrelin was also observed in some malignant cell lines, specifically those negative for ER and PR (11). Likewise, transcription of In1-ghrelin was upregulated 8-folds in high-grade tumor tissue compared to normal breast tissue (12), and was associated with poor survival (59). Furthermore, expression of In1-ghrelin was highly associated with rate of cellular proliferation and expression of GHSR1b, but not with expression of GHSR1a. Unlike association of ghrelin with ER expression, no association between In1-ghrelin and ER was observed (12). Based on these findings, authors postulated that exon 3-deleted preproghrelin and In1-ghrelin may have a more important role in breast cancer progression, compared to native ghrelin.

Similarly, acylated, exon 3-deleted, and In1-ghrelins had different local effects on prostate cancer cell line (36). Overall, In1-ghrelin showed more oncogenic effects compared to native ghrelin.

#### Factors Related to Experiment Setting

Ghrelin has dose-dependent local effects on some cancer cell-lines. The prototype is gastric adenocarcinoma cell-line (47, 78) where a biphasic pattern for stimulatory effects of acylated and des-acylated ghrelins has been observed. While treatment with 1 and 10 nm/l ghrelin increased the proliferation rate, 100 nm/l ghrelin caused an inhibitory effect on proliferation. The stimulatory effect of 1 nm/l ghrelin was weaker than the 10 nm/l dose. Interestingly, plasma ghrelin showed a biphasic prognostic role in patients with gastric adenocarcinoma when it was measured prior to gastrectomy while the tumor tissue is still exposed to ghrelin (134). Patients with the lowest and highest quartiles of pre-operative blood ghrelin had higher rates of survival compared to those with ghrelin levels in the range of 2nd−3rd quartiles. The prognostic pattern of post-operative ghrelin was different. Extrinsic ghrelin had biphasic local effects on breast and pancreas cancer cells, too. In a more general trend suggested by a review, lower doses of ghrelin enhanced proliferation rate and doses >1 μm exerted inhibitory effects on human cancer cell-lines (52). Similarly, baseline serum ghrelin demonstrated a dose-dependent protective role against 10-year risk of esophagus SCC (72). Ghrelin also had dose-dependent effects on angiogenesis. Treatment with lower concentrations increased angiogenesis, while concentrations over 100 nM exerted inhibitory effects (39). Whether in general or just in case of some specific types of cancer, dose/concentration of ghrelin seems to be an important regulatory factor in local and systemic effects of ghrelin.

Another potential regulatory factor related to experiment setting is represented by the constituents of culture media. Using materials such as 4-(2-aminoethyl) benzene-sulphonyl fluoride hydrochloride has been suggested to influence the local effects of ghrelin by interfering in de-acetylation process (136). However, this theory is contradicted by the almost comparable effects of ghrelin with des-acylated ghrelin observed in the studies that investigated them both (29, 47, 75).

Given the de-acylation ability of serum esterase and short half-life of acylated ghrelin in blood stream, it could be postulated that different storage, processing, and analyzing methods of serum samples may result in heterogenous estimates of circulating acylated and des-acylated ghrelins in *in-vivo* and clinical studies. Implementing a standard and consistent protocol for processing [e.g., adding HCL to plasma samples (132)], storage, and measurement of samples can address this issue.

#### Extent of Exposure of Tumor Cells to Local and Systemic Ghrelin in Body

Ghrelin is mainly produced by the stomach and some other organs such as kidney, intestine, and endocrine tissues (2, 5). It could be hypothesized that tumors originating within or in adjacent to these organs may have higher paracrine exposures to ghrelin. Therefore, *in-situ* local effects of ghrelin on tumors originated from these organs may contribute more prominently to the summary effects of ghrelin on carcinogenesis or behavior of the tumor. In support, the prognostic pattern of pre-operative blood ghrelin in patients with gastric cancer (134) was in line with the pattern of ghrelin local effects observed in *in-vitro* studies (47). However, the prognostic role of ghrelin measured after gastrectomy, when tumor cells are no longer exposed to paracrine ghrelin produced by the stomach, was distinctly different and more justifiable by ghrelin systemic effects (134). Similar dominancy of local effects in summary role of ghrelin was observed in case of renal cell carcinoma (85) and colorectal carcinoma (30). Another potential example could be the gastrointestinal stromal tumor (GIST) in stomach (137). However, current data is scarce about contribution of ghrelin to development or prognosis of this cancer. On the other hand, physiologic barriers may limit access of tumors originated in some organs to systemic ghrelin (e.g., CNS tumors).

### Systemic Effects

#### Orexigenic

One of the well-studied systemic effects of ghrelin in body is stimulation of appetite (73). Ghrelin has long been considered as a potential treatment for cancer-associated cachexia (138–140). Improving effects of ghrelin on food intake and body composition have been reported in patients with different types of cancer (120, 140, 141). Regarding documented poor prognostic role of cachexia in cancer patients (142), orexigenic effects of body ghrelin could be considered an invariable positive piece in the puzzle of ghrelin contribution to cancer prognosis, regardless of cancer type.

#### Anti-inflammatory

Chronic inflammation pre-disposes to tumorigenesis in different types of cancers. Local inflammation is linked to tumor microenvironments and cancer progression (40, 41). Ghrelin has both general and site-specific anti-inflammatory effects. In general, it mitigates production of pro-inflammatory cytokines (TNFα, IL-1β, IL-6, and IL-8) and nuclear factor κB. Both ghrelin and des-acylated ghrelin suppress macrophage-originated inflammatory cytokines and expression of COX-2 (143). Furthermore, ghrelin can activate gastric vagus nerve that conveys immune information of the GI tract to the hypothalamus (144, 145). Vagus stimulation reduces macrophage-originated chemokines and attenuates inflammatory response in GI tract mucosa (133, 146). Ghrelin could also attenuate inflammation in epithelial cells of colon (6).

The anti-inflammatory effects of ghrelin can protect against carcinogenesis, specifically in case of inflammation-derived cancers. Ghrelin inhibited carcinogenesis in mouse model of inflammation-associated colorectal cancer (42). At the same study, ghrelin could not prevent carcinogenesis in APC-mutated mice, emphasizing the importance of anti-inflammatory effects of ghrelin in preventing carcinogenesis in inflammation-mediated model. Similarly, ghrelin had inhibitory effects on esophageal Barret's carcinogenesis by opposing inflammation (69).

Furthermore, inflammation contributes to cachexia and cancer metastasis, both of which are notorious prognostic factors in cancer (134). Therefore, the anti-inflammatory effects of ghrelin could be considered an invariable positive contributor to cancer survival, regardless of cancer type. Orexigenic and anti-inflammatory effects of ghrelin could explain better prognosis of gastric cancer patients with post-operative blood ghrelin in range of higher quartiles (134).

#### Regulation of Endocrine Function

Ghrelin regulates some endocrine function with potential contributions to cancer biology. Inhibitory effects of both native ghrelins on expression and activity of aromatase enzyme in adipose stromal cells is the prototype example (136, 144).

Aromatase-mediated production of estrogen in peripheral adipose tissue is the major driver of hormone-dependent breast cancer and its aggressiveness in post-menopausal women (144). This underlines the efficacy of estrogen receptor antagonist or aromatase inhibitors in hormone-dependent breast cancer patients. Based on a similar rationale, ghrelin could be both protective against carcinogenesis and a positive prognostic factor in estrogen-dependent breast cancer. It also could be considered an adjuvant therapy like other novel therapies suggested (147, 148) to reduce toxicity of conventional therapies (149). In support, three cohorts reported positive prognostic significance of ghrelin immunostainings in breast cancer tissue (96, 97, 103). These cohorts consisted of men or women patients with hormone-dependent breast cancer and mean age of women cases was in post-menopausal range.

Another endocrine effect of ghrelin potentially attributable to cancer biology is its stimulation of growth hormone secretion by binding to GHSR1a. GH regulates serum concentrations of IGF1 that has pro-neoplastic effects (150). However, existing evidence does not strongly support contribution of ghrelin-GH-IGF1 axis to cancer development or progression.

### Recommendations for Future *in-vitro* and Clinical Studies

We underscored some potential factors regulating local effects of ghrelin. There is no comprehensive single study on one type of cancer to have investigated all these factors together. This is a limitation for interpreting potential roles of these factors in case of a single cancer. The regulating factors highlighted here should be considered in designing future *in-vitro* studies.

Separate and pooled interpretations of local and systemic effects of ghrelin on each type of cancer and consideration of the role of regulating factors could help evaluate the rational for conducting clinical trials investigating exogenous ghrelin on patients with corresponding cancer.

There is vast heterogeneity in the design of experiments, assessment techniques, and dose and type of materials used. This limits the possibility to compare the results of studies on different cell-lines. The majority of current clinical trials reported short-term effects of ghrelin on appetite and body composition of cancer patients. Longer-term effects of exogenous ghrelin on cancer progress and prognosis would shed further lights on clinical applicability of exogenous ghrelin.

## Conclusions

Current literature shows controversies in attribution of ghrelin to cancer. To address controversial results between local effects of ghrelin on different tumor cells, we attempted to scrutinize factors that could regulate ghrelin local effects. Type of ghrelin receptor, type of ghrelin peptide and its splice variants, dose of ghrelin and other experimental settings, and local access of tumor cells to ghrelin in body were main factors supposed to regulate local effects of ghrelin. To address apparent discrepancies between *in-vitro* and clinical studies of ghrelin attribution to one type of cancer, we delineated local and systemic effects of ghrelin and interpreted the final clinical role as a summary of local and systemic effects. Future *in-vitro* and clinical studies are recommended to consider these approaches in their study design.

## Author Contributions

SS-J, FS, and KZ: concept and design. SS-J, FS, AP, LK, and VR: data-acquisition. SS-J and KZ: supervision. SS-J, FS, AP, and LK: drafting paper. SS-J, FS, AP, LK, VR, and KZ: revising paper and approval to submit.

### Conflict of Interest

The authors declare that the research was conducted in the absence of any commercial or financial relationships that could be construed as a potential conflict of interest.
