# Supplementary material for: Attribution of Ghrelin to Cancer; Attempts to Unravel an Apparent Controversy
Source: Front Oncol. 2019 Oct 16;9:1014. doi: 10.3389/fonc.2019.01014 (PMC6805778; doi:10.3389/fonc.2019.01014)
Supplement: Supplementary file 1 [file Data_Sheet_1.zip › Table 1.docx]

Table S1- Table of evidence for breast cancer

| Reference | Design | Cell line/study group | Intervention | Main Assessment | Main Findings | Mechanism |
| --- | --- | --- | --- | --- | --- | --- |
| Cassoni et al. 2001  (29) | In-vitro | T47D, MDA-MB231, MCF7 | aG (1-2000 nM)  daG (1-2000 nM) | RT-PCR (GHS-R1a)  [3H]-thymidine incorporation  Cell number | *Receptor gene expression (GHSR1a):* All cell lines ⊗  *Cell Proliferation (by aG)*:  MDA-MB231↓, MCF7↓  *Cell Proliferation (by daG)*:  MCF7↓ | Inhibition of cell proliferation |
| Wagner et al. 2006  (67) | Case-control | Cancer (405), Control (460) |  | TaqMan allelic discrimination (*Ghrl* and *Ghsr* SNPs and haplotypes) | *Risk of cancer:*  *Ghrl* SNPs ↔  *Ghrl* haplotypes GGAC and GGAT↓  *Ghsr* SNP Gly57Gly↑ |  |
| Dossus et al. 2008 (66) | Nested case-control | Cancer (1359), Control (2389) |  | TaqMan allelic discrimination (*Ghrl* and *Ghsr* SNPs) | *Risk of cancer:*  *Ghrl* rs171407-G allele↑  *Ghsr* rs2948694-GG genotype ↑ | Interactions between ghrelin, GH, IGF-I, and anthropometrical factors |
| Feigelson et al. 2008 (65) | Nested case-control | Cancer (648), Control (659) |  | Single-base extension (*Ghrl* SNPs) | *Risk of cancer:*  *Ghrl* SNPs ↔ |  |
| Wolf et al. 2006 (64) | Cross-sectional | Cancer (14) |  | RIA (plasma total ghrelin) | *Ghrelin concentration:*  cachectic > noncachectic |  |
| Riezzo et al. 2013 (63) | Cross-sectional | Cancer (35) |  | ELISA (serum total ghrelin) | *Ghrelin concentration (AUC):*  Patients with CADS > patients without CADS |  |
| Al-Khawaja et al. 2015 (60) | Cross-sectional | Cancer (24), benign lesion (16), normal (16) |  | IHC (tissue ghrelin and GHS-R1a) | *Ghrelin peptide:*  Normal ⊗  Benign ⊗  Carcinoma ⊕  *Receptor peptide (GHS-R1a):*  Normal ⊕  Benign ⊕  Carcinoma ⊕  Carcinoma < Normal  *Lymph node involvement:*  Tissue GHS-R1a peptide level↓ |  |
| Sambiasi et al. 2017 (62) | Case-control | Cancer (25), control (38) |  | MIA (plasma ghrelin) | *Risk of cancer:* ↔ |  |
| Gronberg et al. 2012 (58) | Retrospective cohort | Cancer (144) |  | IHC (tissue ghrelin) | *Survival:* Ghrelin peptide level ↑ |  |
| Gronberg et al. 2017 (149) | Retrospective cohort | Cancer (190), control (190) |  | IHC (tissue ghrelin) | *Survival:* Ghrelin peptide level ↑ |  |
| Pellatt et al. 2015 (69) | Retrospective cohort | Cancer (2341) |  | Multiplex bead array assay (*Ghrl* SNPs) | *Survival:*  *Ghrl* rs27647-GG SNP ↓  *Ghrl* rs3755777-C SNP ↑ |  |
| Gronberg et al. 2018 (59) | Retrospective cohort | Male cancer (197) |  | IHC (tissue ghrelin) | *Survival:* Ghrelin peptide level ↑ |  |
| Jeffery et al. 2005 (11) | In-vitro | T47D, MDA-MB-231, MCF7, MDA-MB-435 | aG (0-1000 nM) | RT-PCR (preproghrelin, exon 3-deleted ghrelin, GHS-R1a, GHS-R1b)  Real time RT-PCR (preproghrelin, exon 3-deleted ghrelin)  Wst-1 proliferation assay | *Ghrelin gene expression (preproghrelin):* All cell lines ⊕  *Ghrelin gene expression (exon 3-deleted ghrelin):* All cell lines ⊕  *Receptor gene expression (GHS-R1a):* All cell lines ⊕  *Receptor gene expression (GHS-R1b):* All cell lines ⊕  *Cell Proliferation (by aG)*:  MDA-MB231↑, MDA-MB-435↑  MCF7↔ | Promotion of cell proliferation |
|  | Cross-sectional | Cancer (23), control |  | IHC (tissue aG, exon 3-deleted ghrelin, GHS-R1a and GHS-R1b) | *Ghrelin peptide (aG):*  Normal ⊕  Carcinoma ⊕  Carcinoma > normal  *Ghrelin peptide (exon 3-deleted ghrelin):*  Normal ⊕  Carcinoma ⊕  Carcinoma > normal  Grade 3 > Grade 1  *Receptor peptide (GHS-R1a):*  Normal ⊕  Carcinoma ⊕  *Receptor peptide (GHS-R1b):*  Normal ⊗  Carcinoma ⊕ |  |
| Gahete et al. 2011 (12) | In-vitro | MDA-MB-231 | Transfection with In1-ghrelin | Real time RT-PCR (ghrelin, In1-ghrelin, GHS-R1a, GHS-R1b, GOAT)  Proliferation assay | *Ghrelin gene expression (native ghrelin):* ⊗  *Ghrelin gene expression (In1-ghrelin):* ⊕  *Receptor gene expression (GHS-R1a):* ⊗  *Receptor gene expression (GHS-R1b):* ⊕  *GOAT gene expression:* ⊕  *Cell proliferation (by transfection with In1-ghrelin):* ↑ | Promotion of cell proliferation |
|  | Cross-sectional | Cancer (40), control (4) |  | Real time RT-PCR (ghrelin, In1-ghrelin, GHS-R1a, GHS-R1b, GOAT) | *Ghrelin gene expression (native ghrelin):*  Normal ⊕  Carcinoma ⊕  Carcinoma = Normal  *Ghrelin gene expression (In1-ghrelin):*  Normal ⊕  Carcinoma ⊕  Carcinoma > Normal  *Receptor gene expression (GHS-R1a):*  Normal ⊕  Carcinoma ⊕  Carcinoma = Normal  Native ghrelin and In1-ghrelin ↔  *Receptor gene expression (GHS-R1b):*  Normal ⊗  Carcinoma ⊕  Native ghrelin ↔  In1-ghrelin↑  *GOAT gene expression:*  Normal ⊕  Carcinoma ⊕  Carcinoma > Normal  In1-ghrelin ↑ |  |
| Rincon-Fernandez et al. 2018 (61) | In-vitro | MDA-MB-231, MCF7 | Transfection with ghrelin,  Transfection with In1-ghrelin | Proliferation assay  Invasion/migration assay  Sphere-formation assay | *Cell proliferation (by transfection with ghrelin):*  MDA-MB-231 ↑  MCF7 ↔  *Cell proliferation (by transfection with In1-ghrelin):*Both cell lines ↑  *Cell invasion/migration (by transfection with ghrelin):* Both cell lines ↔  *Cell invasion/migration (by transfection with In1-ghrelin):*  Both cell lines ↑  *Sphere formation (by transfection with ghrelin):* Both cell lines ↔  *Sphere formation (by transfection with In1-ghrelin):*Both cell lines ↑ | Overexpression of MAP/ERK, TGF-β1, JAG1/Notch, and/or Wnt/β-catenin |
|  | Retrospective cohort | Cancer (117) |  | Real time RT-PCR (In1-ghrelin) | *In1-ghrelin gene expression:*  Lymph node involvement↑  Disease-free survival↓ |  |

aG, acyl-ghrelin; AUC, area under the curve; CADS, chemotherapy-associated dyspepsia syndrome; daG, des-acyl ghrelin; ELISA, enzyme-linked immunosorbent assay; GH, growth hormone; GHS-R, growth hormone secretagogue receptor; GOAT, ghrelin-O-acyltransferase; IGF-1, insulin-like growth factor 1; IHC, immunohistochemistry; In1-ghrelin, intron 1-ghrelin; MIA, magnetic immunoassay; mRNA, messenger ribonucleic acid; RIA, radioimmunoassay; RT-PCR, reverse transcriptase-polymerase chain reaction; SNP, single nucleotide polymorphism

⊕, positive expression; ⊗, negative expression; >, higher; <, lower; =, equal; ↑, increased/improved/positive association; ↓, decreased/deteriorated/negative association; ↔, no effect/association
