# Supplementary material for: Attribution of Ghrelin to Cancer; Attempts to Unravel an Apparent Controversy
Source: Front Oncol. 2019 Oct 16;9:1014. doi: 10.3389/fonc.2019.01014 (PMC6805778; doi:10.3389/fonc.2019.01014)
Supplement: Supplementary file 1 [file Data_Sheet_1.zip › Table 2.docx]

Table S2- Table of evidence for colorectal cancer

| Reference | Design | Cell line/study group | Intervention | Main Assessment | Main Findings | Mechanism |
| --- | --- | --- | --- | --- | --- | --- |
| Lien et al. 2016 (34) | In-vitro | HT-29, HCT-15 | Ghrelin (0.1nM - 1µM) | BrdU proliferation assay | *Cell proliferation (by ghrelin):*  Both cell lines ↑ | Activation of GHS-R, Ras, PI3K, Akt, and mTOR signaling pathway |
| Kawaguchi et al. 2015 (42) | In-vivo | C57BL/6 mouse (AOM-DSS inflammation-associated colon carcinogenesis model; APC-mutant cancer model) | aG (3 nmol daily);  *Ghrl* gene deletion | RT-PCR (IL-1β, IL-6, TNF-α, IFN-γ)  IHC (Inflammatory cell markers F4/80 and MPO) | *Tumor number (by aG):*  AOM-DSS model: ↓  APC-mutant model: ↔  *Tumor number (by Ghrl deletion):*  AOM-DSS model: ↔  APC-mutant model: ↔  *Gene expression (by aG):*  IL-1β↓  IL-6, TNF-α and IFN-γ↔  *F4/80 and MPO peptide level (by aG):* ↓ | Suppressing inflammation; Delaying inflammation-driven carcinogenesis |
| Yoshimura et al. 2017 (77) | In-vivo | Colon 26 tumor-bearing BALB/c mouse | GHS-R agonist Z505-hydrochloride (300 mg/kg Bid) |  | *Tumor weight (by Z-505-hydrochloride):* ↔ |  |
| Villars et al. 2017 (76) | In-vivo | Colon 26 tumor-bearing CD2F1 mouse | GHS-R agonist HM01 (10mg/kg daily and  20mg/kg Bid) |  | *Tumor weight (by HM01):* ↔ |  |
| Campa et al. 2010 (78) | Case-control | Czech: cancer (680), controls (593)  Germany: cancer (569), controls (726) |  | TaqMan allelic discrimination (*Ghrl* SNPs) | *Risk of cancer:*  Czech:  *Ghrl* rs27647-T allele ↓  *Ghrl* rs35683-C allele ↓  Germany:  *Ghrl* rs27647-T allele ↔  *Ghrl* rs35683-C allele ↔ |  |
| Murphy et al. 2017 (46) | Nested case-control | Cancer (523), controls (523) |  | RIA (serum total ghrelin) | *Risk of cancer (total ghrelin*)  Overall risk: ↓  Risk of cancer in <10 years of blood draw: ↓  Risk of cancer in 10-20 years of blood draw: ↔  Risk of cancer in >20 years of blood draw: ↑ |  |
| Sundkvist et al. 2018 (70) | Nested case-control | Cancer (60), controls (60) | None | ELISA (plasma total ghrelin) | *Risk of cancer (total ghrelin*)*:*  Risk of cancer in <5 years of blood draw: ↔  Risk of cancer in 10-20 years of blood draw: ↔ |  |
| Wolf et al. 2006 (64) | Cross-sectional | Cancer (26) |  | RIA (plasma total ghrelin) | *Ghrelin concentration:*  cachectic > noncachectic |  |
| D'Onghia et al. 2007 (71) | Cross-sectional | Cancer (29), controls (50) |  | RIA (serum ghrelin) | *Ghrelin concentration:*  carcinoma < normal  stage I > stage III |  |
| Kemik et al. 2010 (72) | Cross-sectional | Cancer (126), controls (38) |  | RIA (serum ghrelin) | *Ghrelin concentration:*  carcinoma < normal |  |
| Nikolopoulos et al. 2014 (73) | Cohort | Cancer (95), controls (39) |  | ELISA (plasma total ghrelin) | *Ghrelin concentration:*  carcinoma > normal  tumor size ↑  tumor grade ↑  tumor stage ↑  Survival ↔ |  |
| Wu et al. 2017 (75) | Retrospective cohort | Cancer (366) |  | Real-time RT-PCR (GHRLOS LncRNA) | *GHRLOS expression:*  Carcinoma < Normal  Lymph node involvement↓  Distant metastasis↓  Survival↓ |  |
| Waseem et al. 2008 (35) | In-vitro | SW-48, RKO | GHS-R antagonist D-[Lys-3]-GHRP6 (1 μM);  anti-ghrelin neutralizing antibody | RT-PCR (ghrelin, GHS-R1a, GHS-R1b)  WB (ghrelin, GHS-R1a, GHS-R1b)  MTT assay  Invasion/Migration assay | *Cell proliferation (by GHS-R antagonist):*  ↓ in both cell lines  *Cell proliferation (by anti-ghrelin neutralizing antibody):*  ↓ in both cell lines  *Cell invasion/migration (by GHS-R antagonist):*  ↓ in both cell lines  *Cell invasion/migration (by anti-ghrelin neutralizing antibody):*  ↓ in both cell lines |  |
|  | Cross-sectional | Cancer (110), controls |  | IHC (tissue ghrelin, GHS-R1a and GHS-R1b)  RIA (plasma ghrelin) | *Ghrelin peptide:*  Normal ⊕  Carcinoma ⊕  Carcinoma > Normal  *Receptor peptide (GHS-R1a):*  Normal ⊕  Carcinoma ⊕  Carcinoma < Normal  *Receptor peptide (GHS-R1b):*  Normal ⊕  Carcinoma ⊕  Carcinoma > Normal  *Tumor stage:*  Ghrelin peptide level↑  GHS-R1a peptide level↓  GHS-R1b peptide level↑  *Ghrelin concentration:*  Cachectic > noncachectic |  |

AOM-DSS, azoxymethane-dextran sodium sulphate; APC, adenomatous polyposis coli; BrdU, bromodeoxyuridine; ELISA, enzyme-linked immunosorbent assay; GHS-R, growth hormone secretagogue receptor; IFN, interferon; IHC, immunohistochemistry; IL, interleukin; mRNA, messenger ribonucleic acid; MPO, myeloperoxidase; mTOR, mammalian target of Rapamycin; MTT, 3-(4,5-dimethylthiazol-2-yl)-2,5-diphenyltetrazolium bromide; PI3K, phosphoinositide 3-kinase; RIA, radioimmunoassay; RT-PCR, reverse transcriptase-polymerase chain reaction; SNP, single nucleotide polymorphism; TNF, tumor necrosis factor; WB, western blotting; aG: acylated ghrelin; GHRLOS LncRNA: ghrelin antisense strand long non-coding RNA

⊕, positive expression; ⊗, negative expression; >, higher; <, lower; ↑, increased/improved/positive association; ↓, decreased/deteriorated/negative association; ↔, no effect/association
