# Supplementary material for: Attribution of Ghrelin to Cancer; Attempts to Unravel an Apparent Controversy
Source: Front Oncol. 2019 Oct 16;9:1014. doi: 10.3389/fonc.2019.01014 (PMC6805778; doi:10.3389/fonc.2019.01014)
Supplement: Supplementary file 1 [file Data_Sheet_1.zip › Table 3.docx]

Table S3- Table of evidence for gastric Cancer

| Reference | Design | Cell line/study group | Intervention | Main Assessment | Main Findings | Mechanism |
| --- | --- | --- | --- | --- | --- | --- |
| Tian PY et al. 2012 (47) | In-vitro | AGS | Ghrelin and daG (1-10-100 nM) | RT-PCR (GHSR-1a/1b)  MTT assay  Flow cytometry | *Receptor gene expression*  *(GHSR-1a):* ⊕  *Receptor gene expression*  *(GHSR-1b):* ⊕  *Ghrelin gene expression:* ⊗  *Cell proliferation (by Ghrelin): ↑*  *Cell proliferation (by daG): ↑* | Ghrelin induces cell proliferation via ERK1/2, Akt signaling. |
| Jiang et al. 2015 (85) | In-vitro | GES-1 | Ghrelin (10, 100, 1000 nM) | MTT assay  Flow cytometry | *Apoptosis (by Ghrelin):*  ↓ | Ghrelin inhibits apoptosis via GHS-R mediated activation of PI3K/AKt. |
| Tian C et al. 2013 (37) | In-vitro | SGC7901, AGS | Ghrelin  (1, 10, 100 nM) | Real-time PCR (GHSR)  MTT assay  Cell invasion assay  Wound-healing assay | *Receptor gene expression (GHSR):* SGC7901 > AGS  *cell proliferation (by ghrelin):* SGC7901 > AGS  *cell migration (by ghrelin):* SGC7901 > AGS | Ghrelin increases cell migration and metastasis via GHS-R/NF-kB/MMP2 signaling; increases proliferation by inducing CDK6 expression and suppressing P53 gene expression |
|  | In-vivo | SGC7901 cell line (overexpressing or naïve ghrelin) grafted to nude mice |  |  | *Tumor size:* Ghrelin over expressing group> control |  |
| Corbetta et al. 2003 (84) | Cross-sectional | Neuroendocrine tumor (6),  Control (35) |  | RIA (total ghrelin in plasma) | *Ghrelin concentration:*  Cancer patients = control |  |
| Isomoto et al. 2005 (83) | Cross-sectional | Cancer (23), control (39) |  | RIA (total ghrelin in plasma) | *Ghrelin concentration:* Gastric cancer < Control  Undifferentiated > Differentiated |  |
| An et al. 2007 (81) | Case-series | Cancer cases:  weight-loss > 5% (27), weight-loss < 5% (53) |  | ELISA (total ghrelin in plasma and tissue) | *Total ghrelin (plasma):*  Before gastrectomy > after gastrectomy  *Total ghrelin (tissue):* tumor < normal  *Total ghrelin (tissue):*  Differentiated > Undifferentiated  *Ghrelin (tissue) ↔ Ghrelin (plasma)* |  |
| Murphy et al. 2012 (49) | Nested case-control | GNCA (261), EGJA (98), Control (441) |  | RIA (total ghrelin in serum) | *Ghrelin concentration:* control > GNCA, EGJA  *GNCA, EGJA risk:*  Ghrelin concentration ↓ |  |
| Zub-Pokrowiecka et al. 2011 (82) | Case-series | Cancer patients with H.pylori infection (25),  Acute/chronic gastritis with H.pylori infection (28),  Healthy H. pylori -negative controls (25) |  | RIA (total ghrelin in plasma)  RT-PCR (GHS-R, ghrelin) | *Total ghrelin (plasma):* before gastrectomy > after gastrectomy  *Receptor gene expression (GHS-R):* Normal mucosa (corpus, fundus) < Gastric cancer  *Ghrelin gene expression:* Normal mucosa (corpus, fundus) > Gastric cancer |  |
| Sadjadi et al. 2013 (48) | Cross-sectional | Non-cardia gastric cancer (72), Cardia gastric cancer (53) |  | RIA (total ghrelin in serum) | *Ghrelin concentration:* control > Non-cardia GC > Cardia GC |  |
| Karaca et al. 2015 (86) | Cohort | Cancer (16) |  | ELISA (total ghrelin in plasma) | *Survival rate:* Pre-radiotherapy ghrelin concentration↑ |  |
| Soleyman-Jahi et al. 2017 (50) | Cohort | Cancer (81) |  | ELISA (total and active ghrelin in plasma) | *Ghrelin concentration (both):*  Pre-gastrectomy > post-gastrectomy  *Survival (total ghrelin)*  Post-gastrectomy ghrelin: ↑  Pre-gastrectomy ghrelin: biphasic  *Survival (active ghrelin)*  Pre-gastrectomy ghrelin: biphasic  Post-gastrectomy ghrelin *↔* |  |

GHS-R, ghrelin hormone receptor; MTT, 3-(4,5-dimethylthiazol-2-yl)-2,5-diphenyltetrazolium bromide; RT-PCR, reverse transcriptase PCR; daG, de-acylated ghrelin; RIA, radioimmunoassay; aG, Acylated ghrelin; ELISA, enzyme linked immunosorbent assay; GNCA, gastric Non-cardia adenocarcinoma; EGJA, esophagogastric junctional adenocarcinoma; ⊕ (positive expression); ⊗ (negative expression); > (higher); < (lower); = (equal); ↑ (increased/improved/positive association); ↓ (decreased/deteriorated/negative association); ↔ (no effect/association)
