# Supplementary material for: Attribution of Ghrelin to Cancer; Attempts to Unravel an Apparent Controversy
Source: Front Oncol. 2019 Oct 16;9:1014. doi: 10.3389/fonc.2019.01014 (PMC6805778; doi:10.3389/fonc.2019.01014)
Supplement: Supplementary file 1 [file Data_Sheet_1.zip › Table 4.docx]

| Table S4- Table of evidence for oesophageal cancer | | | | | | |
| --- | --- | --- | --- | --- | --- | --- |
| Reference | Design | Cell line/study group | Intervention | Main Assessment | Main Findings | Mechanism |
| Konturek et al. 2008 (81) | In-vitro | Barrett (OE-19) cell line,  Barrett’s mucosa tissue | Ghrelin  (1, 20, 150, 200, 450 nM) | FACS (apoptotic genes)  RT-PCR (GHS-R1a in tissue) | *Receptor gene expression (GHSR1a):*  Barrett’s mucosa > normal squamous epithelium  *Apoptosis (by ghrelin):*  OE-19 cells ↔  *TNFa - induced COX-2 (by ghrelin):*  OE-19 cells ↓  *IL-1b expression (by ghrelin):*  OE-19 cells ↓ | Anti-inflammatory effects |
| Mottershead et al. 2007 (89) | Cross-sectional | Adenocarcinoma (22) |  | IHC/ RT-PCR (ghrelin in tissue) | *Ghrelin expression:*  Adenocarcinoma specimens ⊗  Non-neoplastic gastric mucosa ⊕ |  |
| De Martel et al. 2007 (93) | Nested case-control | Adenocarcinoma (31),  Control (79) |  | EIA (Total ghrelin in serum) | *Total ghrelin concentrations:*  Control > case  *Risk of cancer:*  Total ghrelin ↓ (Only in overweight subgroup) |  |
| Doecke et al. 2008 (79) | Case-control | Adenocarcinoma (260), SCC (213),  EGJA (301),  Control (1,352) |  | TaqMan allelic discrimination (*Ghrl* SNPs Rs696217 - Rs4684677) | *Risk of cancer (Ghrl SNP):*  Adenocarcinoma ↔  SCC ↔  EGJA↔ |  |
| Murphy et al. 2011 (49) | Nested case-control | EGJA (98),  Control (441) |  | RIA (Total ghrelin in serum) | *Ghrelin concentrations:*  Control > case  *Risk of cancer:*  Total ghrelin ↓ |  |
| Murphy et al. 2012 (92) | Nested case-control | SCC (82),  Control (82) |  | RIA (Total ghrelin in serum) | *Ghrelin concentrations:*  Control > case  *Risk of cancer:*  Total ghrelin ↓ |  |
| Miyazaki et al. 2012 (45) | Case-series | Cancer (25); subtype not reported |  | ELISA (Total ghrelin in serum) | *Ghrelin concentrations:*  Preoperative > postoperative  *Clinicopathologic features of tumor*:  Total ghrelin ↔ |  |
| Elliott et al. 2019 (94) | Case-series | Cancer (12); both adenocarcinoma and SCC |  | ELISA (total ghrelin in plasma) | *Postoperative ghrelin concentration:*  Decreased from day 10  Recovered by 1 year  *% Weight loss (1 year postoperative):*  Ghrelin concentration ↔ |  |
| Sadjadi et al. 2013 (48) | Case-control | Adenocarcinoma (36), Control (36) /  SCC (59),  Control (59) |  | RIA (Total ghrelin in serum) | *Risk of cancer (total ghrelin)*:  SCC ↓  Adenocarcinoma ↔ |  |
| Omoto et al. 2014 (90) | Cohort | SCC (210) |  | IHC (ghrelin in tumor tissue) | *Ghrelin peptide level:*  Advanced clinical stage > lower stage  Tumor invasion ↑  Pathological stage ↑  Tumor differentiation ↑  Venous invasion ↑  Survival ↔ |  |
| Takata et al. 2015 (43) | Clinical trial | SCC:  Treated (20)  Untreated (20) | Ghrelin IV infusion (3 µg/kg bid or 0.5 µg/kg/h continuous) | SIRS duration  SIRS markers | *SIRS durations:*  Treated group < Untreated group  *C-reactive protein concentrations:*  Treatment < control | Anti-inflammatory effects |

GHS-R, ghrelin hormone receptor; EGJA, esophagogastric junctional adenocarcinoma; IHC, immunohistochemistry; RT-PCR, reverse transcriptase PCR; RIA, radioimmunoassay; ELISA, enzyme linked immunosorbent assay; mRNA, messenger ribonucleic acid; SNP, single nucleotide polymorphism; SIRS, systemic inflammatory response syndrome

⊕ (positive expression); ⊗ (negative expression); > (higher); < (lower); = (equal); ↑ (increased/improved/positive association); ↓ (decreased/deteriorated/negative association); ↔ (no effect/association)
