# Supplementary material for: Attribution of Ghrelin to Cancer; Attempts to Unravel an Apparent Controversy
Source: Front Oncol. 2019 Oct 16;9:1014. doi: 10.3389/fonc.2019.01014 (PMC6805778; doi:10.3389/fonc.2019.01014)
Supplement: Supplementary file 1 [file Data_Sheet_1.zip › Table 5.docx]

Table S5- Table of evidence for lung cancer

| Reference | Design | Cell line/study group | Intervention | Main Assessment | Main Findings | Mechanism |
| --- | --- | --- | --- | --- | --- | --- |
| Ghe et al. 2002 (96) | In-vitro | CALU-1 (non-endocrine) | Ghrelin (1-2000 nM) | RT-PCR (GHS-R1a)  [3H]-thymidine incorporation proliferation assay | *Receptor gene expression (GHS-R1a):* ⊗  *Cell Proliferation (by ghrelin)*: ↔ |  |
| Tsubouchi et al. 2017 (133) | In-vitro | HLC-1 (non-endocrine) | aG (10 μM) | BrdU proliferation assay  MTT assay  TUNEL assay  Mitochondrial respiration assays | *Cell Proliferation (by aG)*: ↔  *Cell viability (by aG):* ↔  *Cell apoptosis (by aG):* ↔  *Mitochondrial respiratory functions (by aG):* ↔ |  |
| Zhu et al. 2018 (101) | In-vitro | A549 (adenocarcinoma) | aG (0.1-10 nM) | BrdU proliferation assay | *Cell Proliferation (by aG)*: ↑ | Upregulating phosphorylated ERK, activating PI3K/Akt/mTOR/P70S6K signaling pathway |
| Li et al. 2019 (98) | In-vitro | H1650 and HCC827/GR (drug-resistant NSCLC),  HCC827 (non-drug resistant NSCLC) | GHSR antagonist (d -lys-GHRP-6).  GHSR siRNA | RT-PCR (GHSR)  MTT assay  Annexin-V cell apoptosis assay  WB (Bcl2, caspase-3, GHSR, p-ERK1/2*,* p-AKT) | *Receptor gene and peptide:*  Drug-resistant cells > non drug-resistant cells  *p-ERK1/2 and p-AKT level:*  Drug resistant HCC827 > HCC827  *p-ERK1/2 and p-AKT level (by GHSR antagonist and GHSR siRNA):* ↓  *Cell proliferation of drug-resistant cells (by GHSR antagonist):* ↓  *Cell apoptosis of drug-resistant cells (by GHSR antagonist):* ↑  *Cleaved caspase-3 level (by* *GHSR antagonist):* ↑  *Bcl2 level (by GHSR antagonist):* ↑ | High activity of GHSR was responsible for the activation  of MAPK/ERK and PI3K/AKT signaling and accompanied cell proliferation in drug-resistant NSCLC cells |
| Tsubouchi et al. 2014 (103) | In-vivo | PTEN-deficient C57BL6/J F6 mouse with urethane-induced lung adenocarcinoma | aG (10 nmol/kg bid) |  | *Animal weight (by aG):* ↑ | Suppressing inflammation, boosting appetite; upregulating phosphorylated Akt and IGF-1 in skeletal muscle fibers, downregulating p38 MAPK, NF-kb, and muscle-specific E3 ubiquitin ligases (Atrogin1 and MuRF1) in skeletal muscle fibers |
| Chen et al. 2015 (102) | In-vivo | C57BL/6J mouse- implanted with Lewis lung carcinoma | aG (0.8 mg/kg bid) |  | *Animal weight (by aG):* ↑  *Tumor weight (by aG):* ↔  *Survival (by aG):* ↑ | Suppressing inflammation, boosting appetite; upregulating phosphorylated Akt, decreasing ubiquitin-proteasome pathway activity, downregulating p38 MAPK/C/EBP-β, reducing Myostatin, and increasing MyoD and Myogenin expression |
| Shimizu et al. 2003 (100) | Cross-sectional | NSCLC (29), small cell lung carcinoma (14), controls (21) |  | RIA (plasma ghrelin) | *Ghrelin concentration:*  Carcinoma = Normal  cachectic > noncachectic |  |
| Karapanagiotou et al. 2009 (99) | Cohort | NSCLC (101), controls (60) |  | ELISA (plasma total ghrelin) | *Ghrelin concentration:*  Carcinoma > Normal  *Survival:*  Ghrelin concentration: ↔ |  |
| Kerenidi et al. 2013 (44) | Cohort | NSCLC (61), small cell lung carcinoma (19), controls (40) |  | RIA (serum total ghrelin) | *Ghrelin concentration:*  Carcinoma > Normal  *Survival:*  Ghrelin concentration: ↔ |  |
| Temel et al. 2016 (105) | RCT | NSCLC;  *ROMANA1:*  Treated (323), Untreated (161)  *ROMANA2:*  Treated (330), Untreated (165) | GHS-R agonist Anamorelin (100 mg daily) |  | *Lean body mass:* ↑  *handgrip strength:* ↔  *Survival:* ↔ |  |
| Takayama et al. 2016 (104) | RCT | NSCLC; 50 mg Anamorelin (65), 100 mg Anamorelin (55), placebo (60) | GHS-R agonist Anamorelin (50 or 100 mg daily) |  | *Lean body mass:* ↔  *handgrip strength:* ↔ |  |
| Katakami et al. 2018 (106) | RCT | NSCLC;  Treated (84), Untreated (90) | GHS-R agonist Anamorelin (100 mg daily) |  | *Lean body mass:* ↑  *Body weight*: ↑  *Appetite:* ↑  *handgrip strength:* ↔  *Fatigue:* ↔  *Survival:* ↔ |  |
| Currow et al. 2017 (106) | RCT | NSCLC;  Treated (345), Untreated (168) | GHS-R agonist Anamorelin (100 mg daily) |  | *Body weight:* ↑ |  |
| Cassoni et al. 2006 (95) | In-vitro | H345 (neuro-endocrine) | aG (10-1000 nM for MTT assay; 100 nM for TUNEL assay)  daG (10-1000 nM) | RT-PCR (ghrelin, GHS-R1a)  IHC (aG/daG)  MTT assay  TUNEL assay | *Ghrelin gene expression:* ⊕  *Ghrelin peptide:* ⊗  *Receptor gene expression (GHS-R1a):*⊗  *Cell proliferation (by aG):* ↓  *Cell proliferation (by daG):*↓  *Cell apoptosis (by aG):*↑ |  |
|  | Cross-sectional | Lung adenocarcinoma (15), lung squamous cell carcinoma (12), lung neuroendocrine tumors (14) |  | RT-PCR (ghrelin, GHS-R1a)  IHC (aG/daG) | *Ghrelin gene expression:*  Adenocarcinoma ⊕  Squamous cell carcinoma ⊕  Neuroendocrine ⊕  *Ghrelin peptide:*  Adenocarcinoma ⊗  Squamous cell carcinoma ⊗  Neuroendocrine ⊕  *Receptor gene expression (GHS-R1a):*  Adenocarcinoma ⊗  Squamous cell carcinoma ⊕  Neuroendocrine ⊕ |  |

aG, acyl-ghrelin; BrdU, bromodeoxyuridine; daG, des-acyl ghrelin; ELISA, enzyme-linked immunosorbent assay; GHS-R, growth hormone secretagogue receptor; IGF-1, insulin-like growth factor 1; IHC, immunohistochemistry; MAPK, mitogen-activated protein kinase; mRNA, messenger ribonucleic acid; MTT, 3-(4,5-dimethylthiazol-2-yl)-2,5-diphenyltetrazolium bromide; RCT, randomized controlled trial; RIA, radioimmunoassay; RT-PCR, reverse transcriptase-polymerase chain reaction; TUNEL, Terminal deoxynucleotidyl transferase dUTP nick end labeling; NSCLC: Non-small cell lung carcinoma

⊕, positive expression; ⊗, negative expression; >, higher; <, lower; =, equal; ↑, increased/improved/positive association; ↓, decreased/deteriorated/negative association; ↔, no effect/association
