# Supplementary material for: Attribution of Ghrelin to Cancer; Attempts to Unravel an Apparent Controversy
Source: Front Oncol. 2019 Oct 16;9:1014. doi: 10.3389/fonc.2019.01014 (PMC6805778; doi:10.3389/fonc.2019.01014)
Supplement: Supplementary file 1 [file Data_Sheet_1.zip › Table 6.docx]

Table S6- Table of evidence for other cancers

| Reference | Design | Cell line/study group | Intervention | | Main Assessment | Main Findings | Mechanism |
| --- | --- | --- | --- | --- | --- | --- | --- |
| Prostate cancer | | | | | | | |
| Jeffery et al. 2002 (11) | In-vitro | DU145, LNCaP, PC3, ALVA41 | Ghrelin (0.1, 1, 5, 10, 20 nM) | | RT-PCR (ghrelin,  GHS-R1a, GHS-R1b)  MTT assay | *Ghrelin gene expression:*  Normal prostate cell line ⊗ Prostate cancer cell lines ⊕  DU145>ALVA41>PC3, LNCaP  *GHS-R1a gene expression:*  Normal prostate cell line ⊕ Prostate cancer cell lines ⊕  ALVA41> PC3>LNCaP, DU145  *GHS-R1b gene expression:*  Normal prostate cell line ⊗ Prostate cancer cell lines ⊕  PC3 > ALVA41, DU145 >LNCaP  *Cell proliferation (by ghrelin)*:  PC3 ↑ | Ghrelin induces proliferation  via binding to GHS-R 1a |
| Maugham et al. 2018 (111) | In-vivo | PC3 cells were grafted into B6.SVJ129 Rag1^-/-^ mice | daG (100 µg/Kg) | | IHC (Ki67, CD31) | *daG administration effects:*  Tumor volume *↔*  Tumor Ki67 expression *↔*  Tumor CD31 expression *↔* |  |
| Maugham et al. 2018 (112) | In-vitro | PC3 | GHSR antagonist [D-Lys 3]-  GHRP-6 (0-10 µM) | | Wst-1 and Incucyte Zoom cell proliferation assays | *Cell proliferation (by GHSR antagonist):* *↔* |  |
|  | In-vivo | PC3 cells were grafted into NOD/SCID mice | GHSR antagonist [D-Lys 3]-  GHRP-6 (20 nmol/mouse) | | qPCR (EGFR)  IHC (Ki67) | *Tumor volume and weight (by GHSR antagonist): ↓*  *EGFR expression in tumor (by GHSR antagonist): ↓*  Resistant to GHSR antagonist effects by 18 days  Tumor Ki67 expression (by GHSR antagonist): *↔* |  |
| Cassoni et al. 2004 (11) | In-vitro | DU145, PC3, LNCaP | aG, daG (10-100 nM) | | RT-PCR (ghrelin, GHS-R1a, GHS-R1b)  IHC (ghrelin, GHS-R1a, GHS-R1b)  EIA (aG)  [3H]thymidine into DNA (cell proliferation assay) | *Ghrelin gene expression:*  PC3 ⊕ DU145, LNCaP ⊗  *Ghrelin peptide level*:  PC3 ⊕ DU145, LNCaP ⊗  *GHS-R 1a/1b gene expression*:  DU-145 ⊕, LNCaP ⊗, PC3 ⊗  *Ghrelin concentration:*  PC3 ⊗, LNCaP ⊗, DU-145 ⊗  *Cell proliferation (by aG & daG)*:  DU-145 ↓  PC3 biphasic effect  LNCaP ↔ |  |
|  | Cross-sectional | Benign (10), PC (10) |  | | RT-PCR (ghrelin, GHS-R1a, GHS-R1b)  In-situ hybridization  IHC (ghrelin, GHS-R1a, GHS-R1b) | *Ghrelin gene expression:*  Carcinomas ⊕ Benign hyperplasia ⊕  *Ghrelin peptide level:*  Carcinomas ⊗ Benign hyperplasia ⊗  *GHS-R 1a/1b gene expression*:  Carcinomas ⊗  *GHS-R 1b gene expression*:  Benign hyperplasia ⊕ |  |
| Yeh et al. 2005 (36) | In-vitro | PC3, LNCaP | daG,  exon-3 deleted ghrelin  (0.1, 1,10, 100, 1000 nM) | | RT-PCR (aG and exon-3 deleted ghrelin)  MTT assay | *aG & exon-3 deleted ghrelin gene expression*: PC3⊕  LNCaP⊕  PC3 > LNCaP  *Cell proliferation (by aG):*  PC3↑ LNCaP↑  PC3 ↔ LNCaP  *Cell proliferation (by exon-3 deleted preproghrelin)* PC3⊗, LNCaP ⊗ | Ghrelin Induces proliferation via MAPK (ERK1,2) activation |
|  | Cross-sectional | Benign (11), PC (26) |  | | IHC (ghrelin, exon-3 deleted preproghrelin*)* | *Ghrelin peptide level:*  Cancer > benign  *Exon-3 deleted preproghrelin peptide level:*  Cancer > benign |  |
| Mungan et al. 2008 (114) | Cross-sectional | PC (30), Benign (50) |  | | EIA (total ghrelin in serum) | *Ghrelin concentration:* PC = benign |  |
| Malendowicz et al. 2009 (113) | Cross-sectional | PC (18), Benign (12), Control (16) |  | | EIA (total ghrelin and aG in serum) | *aG concentration:* PC > benign & control  *Total ghrelin concentration:*  PC = Benign |  |
| Gomez-Gomez et al. 2018 (115) | Cross-sectional | PC (183),  Control (129) |  | | ELISA (plasma GOAT) | *GOAT concentration:*  Cancer > control  High Gleason-score patients > low  Gleason-score patients  Gleason score ↑  Presence of metastasis at the time of diagnosis ↑ |  |
| Hormaechea  et al. 2017 (108) | In-vitro | RWPE-1 (normal prostate cell line), 22Rv1, LNCaP, VCaP, PC-3 and  DU145 | In-1 ghrelin, aG | | Migration assay (wound healing assay)  Proliferation assay (MTT)  RT-PCR (In-1 ghrelin and aG)  WB (p-ERK1/2) | *Ghrelin mRNA expression:*  LNCaP > VCaP > RWPE-1> 22Rv1> *PC3 >* DU145  *In-1 ghrelin mRNA expression:*  *PC3 >* DU145 > VCaP, 22Rv1, LNCaP > RWPE-1  *Cell proliferation (by aG):*  RWPE-1 ↔  *PC3, 22Rv1* ↑  LNCaP, VCaP, RWPE-1, DU145↔  *Cell proliferation* (by *In-1 ghrelin*):  RWPE-1 ↔  22Rv1, VCaP & LNCaP, PC-3, DU145 ↑  *Migration (by In-1 ghrelin)*  ↑ PC3  *Migration (by aG)*  ↔ PC3  *ERK1/2 phosphorylation (by aG & In-1 ghrelin)*  *PC3 & LNCaP* ↑ | Ghrelin induces ERK1,2 signaling and oncogene expression |
|  | In-vivo | PC3 cells were grafted into BALB/cAnNRj mice (10) | PC3 cells were stably-transfected with aG or In-1 ghrelin | |  | *Tumor size:*  In-1 ghrelin transfected graft > aG transfected graft |  |
|  | Cross-sectional | PC (52), control (12) |  | | qPCR (tissue In-1 ghrelin, ghrelin) | *In-1 ghrelin gene expression*:  Patient > control  *Ghrelin gene expression*:  Patient = control  *GHSR1a/1b gene expression:* ⊗  *Tumor progression markers:*  In-1 ghrelin gene expression↑  Ghrelin gene expression↔ |  |
|  |  | PC (30),  Control (20) |  | | ELISA, RIA (aG, In-1 ghrelin in plasma) | *In-1 ghrelin concentration*:  Patient > control  *aG concentration*:  Patient = control |  |
| Clear cell subtype of renal cell carcinoma (ccRCC) | | | | | | | |
| Lin et al. 2015 (119) | In-vitro | 786–0, ACHN,  A-498, 769-P, A-704 | Ghrelin (90,180, 360 nM) | | WB (ghrelin, GHS-R1a)  Migration assay | *Ghrelin peptide level:* 786-0 > 769-P > A-704> ACHN > A-498  *Receptor peptide level (GHS-R 1a):*  769-P > A-498 > 786-0 > ACHN > A-704  *Cell Migration (by ghrelin):* ACHN > A-489 > 786-0 | Activation of PI3K/Akt pathway leads to Snail expression and decreases E-cadherin, which promotes metastasis and regulates endothelial mesenchymal transforming process in metastasis. |
|  | In-vivo | 786-0 cells were grafted into Node-SCID mice | Ghrelin knock-out; ghrelin-naïve | |  | *Metastatic lung nodule numbers:* ghrelin KO group < ghrelin present group |  |
|  | Cohort | ccRCC (562) |  | | Microarray  IHC (ghrelin) | *Ghrelin peptide level:* tumor tissue > normal tissue  *Tumor progression*  Ghrelin ↑  *Survival probability:*  Ghrelin ↓ |  |
| Lin et al. 2019 (120) | Cancer cell line encyclopedia | 15 ccRCC cell lines |  | | Integrity pathway analysis (IPA) | *High ghrelin-expressing group*:  Cellular movement pathway ↑  Aurora A ↑  *Aurora* *A expression*:  Ghrelin expression ↑ |  |
|  | In-vitro | A panel of ccRCC cell lines | Lentivirus-mediated ghrelin over-expression  siRNA (Aurora A,  MMP10, or  GHSR1a) | | RT-PCR (Ghrelin, Aurora A)  WB (Ghrelin, GHSR1a, Aurora A, MMP10)  Trans-well invasion assay | *Baseline ghrelin peptide level:*  Baseline Aurora A peptide ↑  *Aurora A gene and peptide level (by ghrelin overexpression):* ↑  *Cell migration (by ghrelin overexpression):* ↑  *Cell migration (by Aurora A siRNA):* ↓  *Cell migration (by MMP10 siRNA):* ↓  *MMP10 expression* (*by Aurora A siRNA):* ↓  *MMP10 expression* (*by GHSR1a siRNA):* ↓ | Importance of  ghrelin-GHSR1a-Aurora A-MMP10 signaling pathway in ccRCC metastasis |
|  | In-vivo | IV injection of NSG mice with ACHN and 786-0 cancer cells | Ghrelin over-expression and Aurora A silencing in cells | |  | *Lung metastatic nodules:*  Aurora A silenced group < Aurora A-naïve group | Aurora A is required in ghrelin-mediated metastasis |
|  | TCGA database | ccRCC (562) |  | | IHC (Ghrelin, Aurora A) | *Survival probability:*  Aurora A ↓  Ghrelin ↓ |  |
| Ovarian cancer | | | | | | | |
| Bai et al. 2016 (125) | In-vitro | HO-8910 | | Ghrelin (121, 152, 182, 212, 242 nM) | RT-PCR (GHS-R)  MTT assay | *Receptor expression (GHS-R):* HO-8910 ⊕  *Cell proliferation (by Ghrelin):* HO-8910 ↓ | Ghrelin/GHSR decreases cell growth through ERK-dependent pathway |
| Nurkalem et al. 2012 (126) | Cross-sectional | Benign (20), borderline (7), malignant (20) | |  | IHC (total ghrelin) | *Mean score of total ghrelin level:* benign > borderline> malignant  *Rate of moderate-severe expression (%):*  benign < borderline< malignant |  |
| Endometrial cancer | | | | | | | |
| Fung et al. 2013 (127) | In-vitro | Ishikawa, KLE (intact, GHSR1a-knock-out) | | aG, daG (10, 100, 1000 nM) | CyQUANT NF Cell Proliferation assay  MTT assay | *Cell proliferation (with/without ghrelin treatment):*  GHSR-1a knock-out < scrambled control | aG enhances cell proliferation via GHSR1a |
|  | In-vivo | GHSR-1a knock-out Ishikawa cells grafted to NOD/SCID mice | |  | IHC (ki67) | *Tumor size:* GHSR-1a knock-out tumor < scrambled control tumor  *Tumor tissue Ki67:*  GHSR-1a knock-out tumor < scrambled control tumor |  |
|  | Cross-sectional | Normal (5), Different types of endometrial cancer (70) | |  | IHC (ghrelin and GHSR1a) | *Ghrelin and GHSR1a peptide level:*  Normal tissue ⊕  Cancerous tissue ⊕  *Tumor grade:*  Ghrelin ↔  GHSR1a ↔ |  |
| Younes et al. 2015 (128) | Cross-sectional | Carcinoma (55),  Hyperplasia (26) | |  | IHC (total ghrelin) | *Total ghrelin peptide level:* Carcinoma < hyperplasia  Tumor grade ↓  Tumor stage ↔  Lymph vascular invasion ↔ |  |
| Central nervous system cancers | | | | | | | |
| Dixit et al. 2006 (121) | In-vitro | Astrocytoma: CCF-STTG1,  U-87, U-118, SW1088 | | aG (100, 200, 300 ng/ml) | Real-time PCR (Ghrelin, GHSR-1a)  Motility and invasion assays | *Receptor gene expression:* SW1088 > CCF-STTG1 > U87> U118 > NHA  *Cell invasion* *(by aG)* ↑  *Cell migration (by aG)* ↑ SW1088 > U87 > CCF-STTG1 > > U118 | Ghrelin secreted by astrocytoma cells enhances cell motility via autocrine loop (ghrelin-GHSR) |
| Chen et al. 2011 (122) | In-vitro | Glioma: C6 (rat cell line), U251(human cell line) | | Ghrelin (3, 10, 30 nM) | WB (GHS-R)  RT-PCR (GHSR-1a, GHSR-1b)  Migration assay  MTT assay | *Receptor peptide level (GHS-R):* C6 ⊕  U251 ⊕  *Receptor gene expression (GHS-R1a/b):* C6⊕  U251 ⊕  U251> C6  *Cell migration (by ghrelin):* C6, U251 ↑  U251> C6 | Ghrelin increases cell migration via signaling pathways such as NF-KB;  “NF-kB-DNA binding activity” |
| Okada et al. 2016 (123) | Cohort | Glioblastom (39), anaplastic astrocytoma (13), diffuse astrocytoma (11) | |  | IHC (total ghrelin, GHSR-1a) | *Tumor cell proliferation*:  Ghrelin/GHS-R1a ↑ (in  Anaplastic astrocytoma  & Glioblastomas)  *Survival:* high-score ghrelin/GHSR-1a group < low-score ghrelin/GHSR-1a group |  |
| Rozza-de-Menezes et al. 2017 (124) | Cross-sectional | Neurofibromatosis cases (55) | |  | IHC, microarray of neurofibroma tissue (GHS-R expression) | GHS-R expression ⊕  *Tumor size:*  GHS-R expression ↑ |  |
| Oral squamous cell carcinoma (SCC) | | | | | | | |
| Kraus et al. 2016 (129) | In-vitro | BHY, HN | aG (10, 100, 1000 nM) | | RT-PCR (ghrelin, GHSR-1a/1b)  Proliferation assay | *Receptor gene expression (GHSR-1a):* BHY⊕  HN ⊕ BHY> HN  *Receptor gene expression (GHSR-1b):* BHY ⊕ HN ⊕ HN > BHY  *Ghrelin gene expression:* HN ⊕ BHY ⊕ HN > BHY  *Cell proliferation (by aG):* HN ↑ BHY ↑ HN > BHY | aG promotes proliferation via GSK-3b/β-catenin  pathway and up-regulation of cyclin D1  and c-myc |
| Alnema et al. 2010 (130) | Cross-sectional | Benign (10), microinvasive SCC (10), well-differentiated (7), poorly-differentiated (7) |  | | IHC, RIA (tissue ghrelin) | *Ghrelin concentration:* benign > malignant  *Ghrelin concentration*  Well differentiated > Poorly differentiated |  |
| Pancreatic cancer | | | | | | | |
| Duxbury et al. 2003 (38) | In-vitro | PANC1, MIAPaCa2  BxPC3  and Capan2 (well-differentiated cell-lines) | Ghrelin (1, 10, 100 nM) | | RT-PCR (ghrelin, GHSR-1a/1b)  WB (Ghrelin, GHSR-1a/1b)  Invasion assay  Motility assay  MTT assay | *Ghrelin gene expression:* PANC1 ⊕,  MIAPaCa2, BxPC3, Capan2, ⊗  *Ghrelin peptide level:*  PANC1, MIAPaCa2, BxPC3, Capan2 ⊗  *Receptor gene expression (GHS-R1a):* Capan2, MIAPaCa2, PANC1, BxPC3 ⊕  *Receptor gene expression (GHS-R1b):* PANC1, MIAPaCa2, BxPC3, Capan2 ⊕  *Receptor peptide level (GHS-R1a):*  Capan2, PANC1, BxPC3, MIAPaCa2 ⊕  *Receptor peptide level (GHS-R1b):* BxPC3, Capan2, MIAPaCa2, PANC1 ⊕  *Cell proliferation (by ghrelin): ↑*  PANC1 >MIAPaCa2 > BxPC3 > Capan2  Ghrelin dose: 10nM  *Invasion (by ghrelin): ↑*  PANC1 >MIAPaCa2 > BxPC3 > Capan2  *Motility (by ghrelin): ↑*  PANC1 >MIAPaCa2 > BxPC3 > Capan2 | PI3-K/Akt pathway plays an important role in stimulatory effects of ghrelin on migration and invasiveness of pancreatic adenocarcinoma cells. |
| Nanashima et al. 2016 (116) | In-vivo | MIAPaCa2 cells were grafted to BALB/c-nu/nu mice | Compound human ghrelin (30 nM/kg) | |  | *Effects of ghrelin treatment:*  Body weight ↔  Tumor size ↓ |  |
| Corbetta et al. 2003 (84) | Cross-sectional | Pancreatic neuroendocrine tumor (24), Control (35) |  | | RIA (total ghrelin in plasma) | *Ghrelin concentration:* cancer patients = control |  |
| Ekeblad et al. 2007 (80) | Retrospective Cohort | Neuro-endocrine cancer (31),  Control (5) |  | | IHC (total ghrelin, ghrelin receptor)  RT-PCR (ghrelin and ghrelin receptor in tissue)  RIA (total ghrelin in plasma) | *Ghrelin peptide* ⊕  *Ghrelin gene* ⊕  *Ghrelin receptor peptide* ⊕  *Ghrelin receptor gene* ⊕  *Plasma ghrelin:*  Patients = controls  *Survival:*  Tissue ghrelin ↔ Co-expression of ghrelin and its receptor ↔ |  |
| Miura et al. 2018 (117) | Cohort | Treatment-naïve advanced cancer (92) |  | | ELISA (aG and total ghrelin in plasma) | *Ratio of aG/total ghrelin*:  Anorexia ↓  Survival ↔ |  |

daG, de-acylated ghrelin; RT-PCR, reverse transcriptase PCR; WB, Western blotting; MTT, 3-(4,5-dimethylthiazol-2-yl)-2,5-diphenyltetrazolium bromide; PC, Prostate cancer; aG, Acylated ghrelin; EIA, Enzyme immunoassay ; GHS-R, ghrelin hormone receptor; BPH, Benign prostate hyperplasia; IHC, immunohistochemistry; RIA, radioimmunoassay; ELISA, enzyme linked immunosorbent assay

⊕ (positive expression); ⊗ (negative expression); > (higher); < (lower); = (equal); ↑ (increased/improved/positive association); ↓ (decreased/deteriorated/negative association); ↔ (no effect/association)
